# Orientation Engineering of MXene Flakes

**DOI:** 10.1002/adma.72793

**Published:** 2026-03-30

**Authors:** Yizhou Wang, Shuo Li, Tianchao Guo, Dekang Zhu, Ning Chu, Noor Albar, Dana Alsulaiman, Husam N. Alshareef

**Affiliations:** ^1^ Center for Renewable Energy and Storage Technologies (CREST) King Abdullah University of Science and Technology (KAUST) Thuwal Saudi Arabia; ^2^ Materials Science and Engineering Physical Science and Engineering Division King Abdullah University of Science and Technology (KAUST) Thuwal Saudi Arabia

**Keywords:** MXene, MXene devices, MXene flakes, MXene orientation, orientation control, orientation engineering

## Abstract

MXene, a family of 2D transition‐metal carbides, nitrides, or carbonitrides, uniquely integrates metallic conductivity, surface tunability, and compositional versatility, enabling their widespread applications across energy, electronics, sensors, and environmental systems. Beyond conventional optimization (e.g., composition, termination, defects), a new research frontier has recently emerged that focuses on engineering the orientation of MXene flakes, i.e., the deliberate control of flake alignment within MXene‐based structures such as fibers, films, membranes, and gels. Engineered orientation transforms inherently disordered MXene assemblies into highly anisotropic structures with directionally optimized electron, ion, and stress transport pathways. Compared with their randomly stacked counterparts, these oriented MXene structures exhibit remarkably enhanced electrical, ionic, and mechanical properties, unlocking new routes to tailor the macroscopic performance of MXene‐based devices. This review aims to provide a comprehensive and systematic summary of the orientation engineering of MXene flakes, covering the fundamental properties governed by MXene flake orientation, characterization techniques for revealing MXene orientation information, fabrication methodologies for achieving engineered MXene orientation, representative advances of oriented MXene structures in diverse functional systems, and forward‐looking design opportunities for MXene orientation engineering. This article offers both a concise knowledge framework and forward‐looking insights for inspiring the rational design of next‐generation MXene‐based materials and devices.

## Graphical abstract

Orientation engineering of MXene flakes refers to the transformation of disordered MXene flakes into ordered architectures with optimized multiphysical transport properties. Focusing on this topic, this review outlines key principles, characterization, fabrication strategies, and advanced applications of oriented MXene structures. We believe it can offer insights for designing future high‐performance MXene‐based materials and devices.

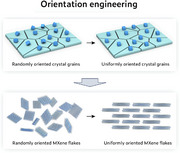

## 1 Introduction

**FIGURE 1:**
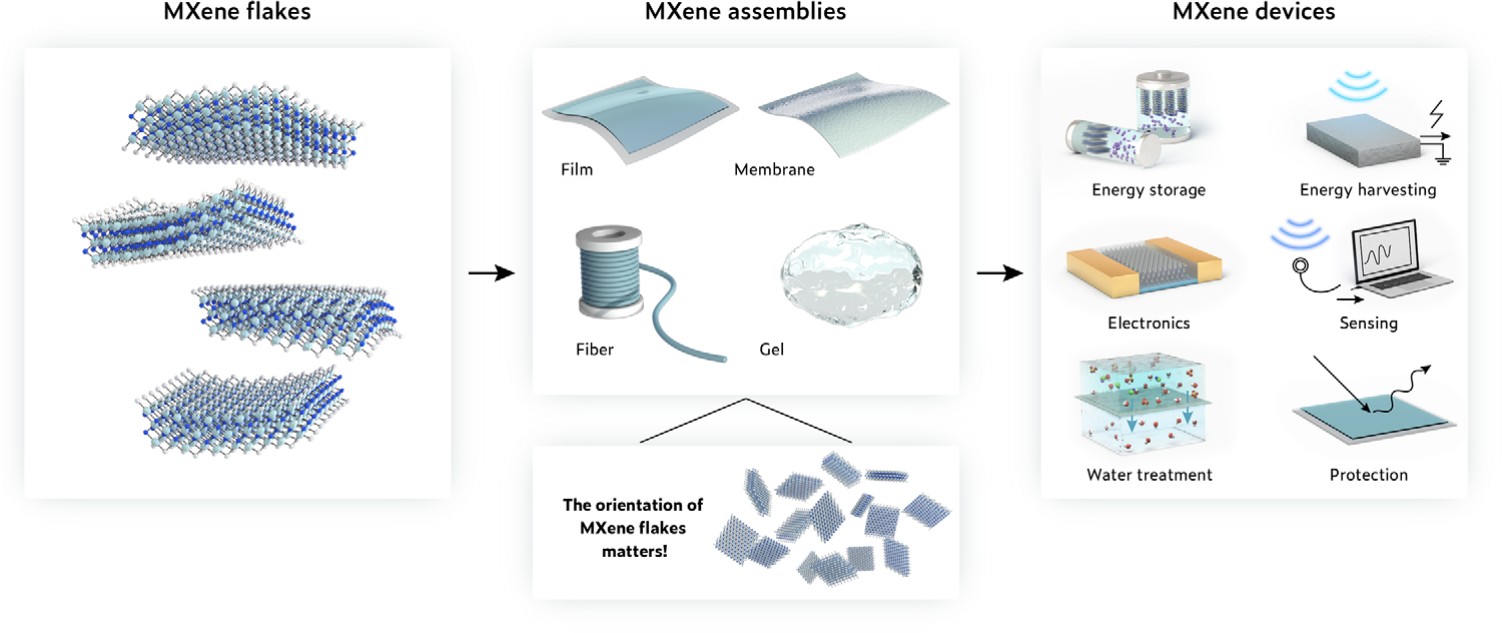
Schematic illustration showing the hierarchy from MXene flakes to assemblies to devices, highlighting the key role of MXene flake orientation.

MXene, a family of 2D transition metal carbides, nitrides, and carbonitrides, is an emerging material first discovered in 2011 [1, 2]. MXene offers a unique set of properties, including metallic conductivity, abundant tunable surface terminations, hydrophilicity, mechanical robustness, and flexibility, which together make it one of the most promising materials for the next generation of energy, electronics, sensing, and environmental technologies [3, 4, 5, 6, 7, 8]. In practical implementations, MXene‐based devices are generally not constructed from a single isolated flake but rather from assembled MXene architectures, such as thin films, membranes, fibers, and gels, in which numerous MXene flakes are integrated together into continuous and processable macroscopic forms (Figure 1). Within these MXene assemblies, the spatial orientation of individual flakes governs key structure–property relationships and profoundly influences the overall performance of the MXene devices. Therefore, understanding the concept of MXene flake orientation and precisely engineering it within macroscopic MXene structures are crucial for optimizing the overall performance of functional MXene devices.

**FIGURE 2:**
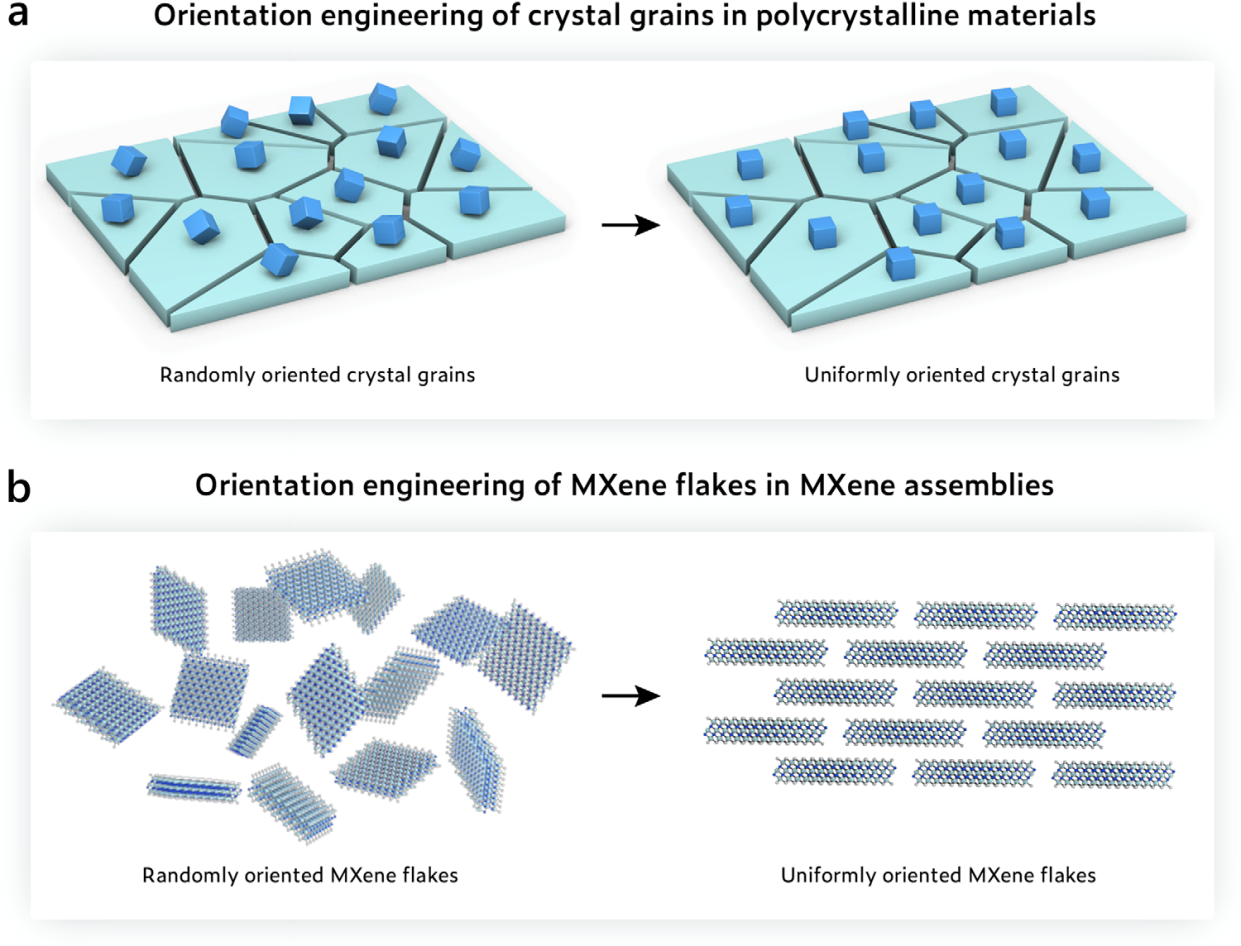
Schematic illustration showing the concept for orientation engineering of (a) crystal grains in polycrystalline materials and (b) MXene flakes in MXene assemblies.

The concept of orientation engineering in MXene assemblies can be effectively rationalized by drawing an analogy with polycrystalline materials [9]. In such material systems, individual grains act as the fundamental structural units. When these grains are randomly oriented, their varying crystallographic directions lead to spatially heterogeneous properties and disrupt the continuity of electron and ion transport across grain boundaries. In contrast, engineering different grains along a preferred orientation can enhance structural coherence and facilitate more efficient multiphysical transport, ultimately improving the macroscopic performance of the polycrystalline material (Figure 2a). Analogously, MXene flakes in assembled architectures can also be regarded as orientation‐dependent structural units, where each flake functions akin to a crystallographic grain. The collective performance of the MXene assembly is thus intimately linked to the orientation of individual flakes. Random orientation of MXene flakes, i.e., misalignment of basal planes, leads to interfacial disorder and even large voids, which compromise the structural integrity and overall performance of MXene assemblies [10, 11]. Engineering the orientation of MXene flakes into highly ordered architectures strengthens interflake coupling and promotes more continuous and directional multiphysical transport across adjacent flakes, thereby enabling significant improvements in the macroscopic performance of the assembled structure (Figure 2b).

In addition to widely used MXene optimization routes that focus on chemistry and composition (e.g., surface terminations, interlayer chemistry, and composite formulation), orientation engineering represents a distinct and generalizable strategy because it regards flake orientation as an independent structural parameter that can be tuned without necessarily changing MXene chemistry. The orientation engineering strategy reshapes transport pathways in MXene assemblies and thus provides a general design framework applicable to MXene thin films, membranes, fibers, and gels, which can further guide device design based on them. Because of this, in recent years, more focus has been given to the critical role of flake orientation in MXene‐based materials [12, 13, 14, 15]. With the growing interest in this emerging area, there is a need for a timely and comprehensive framework to understand how orientation affects functionality and how it can be rationally engineered. Herein, we present a critical overview of the principles, strategies, and recent advances in controlling MXene flake orientation. We first discuss how the orientation of MXene flakes governs the anisotropic properties of MXene assemblies, along with current methodologies for their orientation characterization. We then categorize and examine various orientation engineering strategies, including spontaneous self‐assembly and externally guided approaches. Furthermore, we highlight how rational orientation design can be leveraged to enhance the performance of MXene‐based devices across a broad range of applications, including electronics, energy storage, sensing, actuation, and beyond. Finally, we outline key challenges and emerging opportunities in this rapidly growing field.

## 2 Fundamentals of MXene Orientation Engineering

### 2.1 Basics of MXene

**FIGURE 3:**
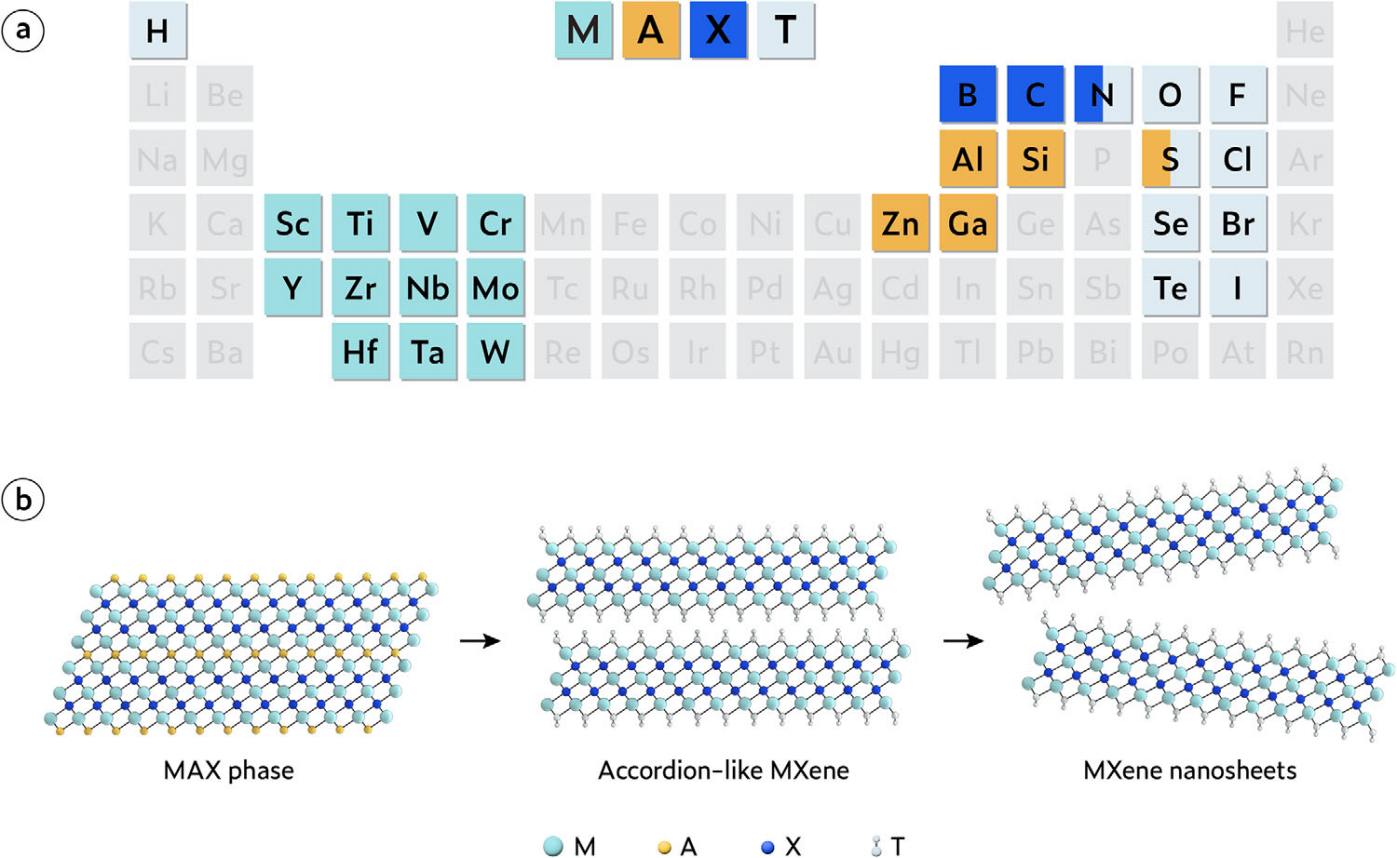
Basic information of MXene. (a) Periodic table highlighting the representative “M,” “A,” and “X” elements constituting MAX phases. (b) Schematic illustration of the selective etching and delamination process transforming MAX phases into 2D MXene.

MXene is a family of 2D transition‐metal carbides, nitrides, and carbonitrides derived from its parent MAX phases [1]. MXene is produced by selectively removing the A layer from MAX phases, and its graphene‐like 2D morphology has therefore led to the adoption of the “‐ene” suffix. MXene and its parent MAX phase have the general formula of M*_n_*_+1_X*_n_* T*_x_* and M*_n_*_+1_AX*_n_*, respectively, where M represents a transition metal such as Ti, Mo, V, or Nb, A is typically an element from groups 13 or 14 such as Al or Si, X is usually carbon or nitrogen, and T represents the surface terminations such as ─O, ─OH, and ─F (Figure 3a).

MXene is typically synthesized through a selective etching process, where the layered parent MAX phases are chemically treated to remove the “A” layer, resulting in the formation of 2D MXene materials (Figure 3b). This process involves the use of etchants, such as hydrofluoric acid (HF) or fluoride‐containing solutions (e.g., LiF + HCl), which selectively dissolve the “A” element from the MAX phase, leaving behind the layered M*_n_*_+1_X*_n_* structure with abundant fluorine and oxygen‐containing surface functional groups. Taking the first discovered and most widely studied Ti_3_C_2_T*_x_* MXene as an example, the reaction during the etching process can be indicated by the following equation: Ti_3_AlC_2_ + 3HF → Ti_3_C_2_T*_x_* + AlF_3_ + 3/2 H_2_ [16]. This process yields multilayered accordion‐like MXene, in which single‐layer MXene flakes are still loosely stacked by van der Waals forces. To separate the MXene flakes from the accordion‐like structure, an additional delamination process is typically required using intercalation agents, including inorganic cations (e.g., Li^+^), organic cations (e.g., tetrabutylammonium cation or tetramethylammonium cation), and neutral molecules (e.g., dimethyl sulfoxide) [17, 18, 19, 20]. Considering the high safety risks posed by the strong toxicity and corrosiveness of HF, researchers have developed a variety of alternative etching methods to reduce experimental hazards and improve the safety of MXene preparation. These new methods include molten salt etching, alkali etching method, and chemical vapor deposition [21]. These alternative methods not only improve the safety of MXene preparation but also provide more possibilities for tuning the structure and properties of MXene [22].

While the first discovered Ti_3_C_2_T*_x_* MXene remains a focal point of research, a wide variety of novel MXene have since been synthesized and extensively investigated. These novel MXene types encompass a diverse range of structural and compositional variations, including different metal elemental compositions (such as Ti‐based, Mo‐based, and high‐entropy‐based), various numbers of constituent unit layers (2, 3, 4, and 5 layers), and distinct surface functional groups (e.g., ─F, ─O, ─S, ─Se) [23, 24, 25]. The number of MXene species predicted by theoretical calculations even far exceeds the variety that has been experimentally synthesized to date [26]. Since significant performance variations exist among different types of MXene, precisely tailoring the elemental composition, functional groups, and structural parameters of MXene can achieve diverse material characteristics and performance, enabling the optimization of MXene in different application scenarios.

### 2.2 Orientation‐Dependent Properties of MXene

**FIGURE 4:**
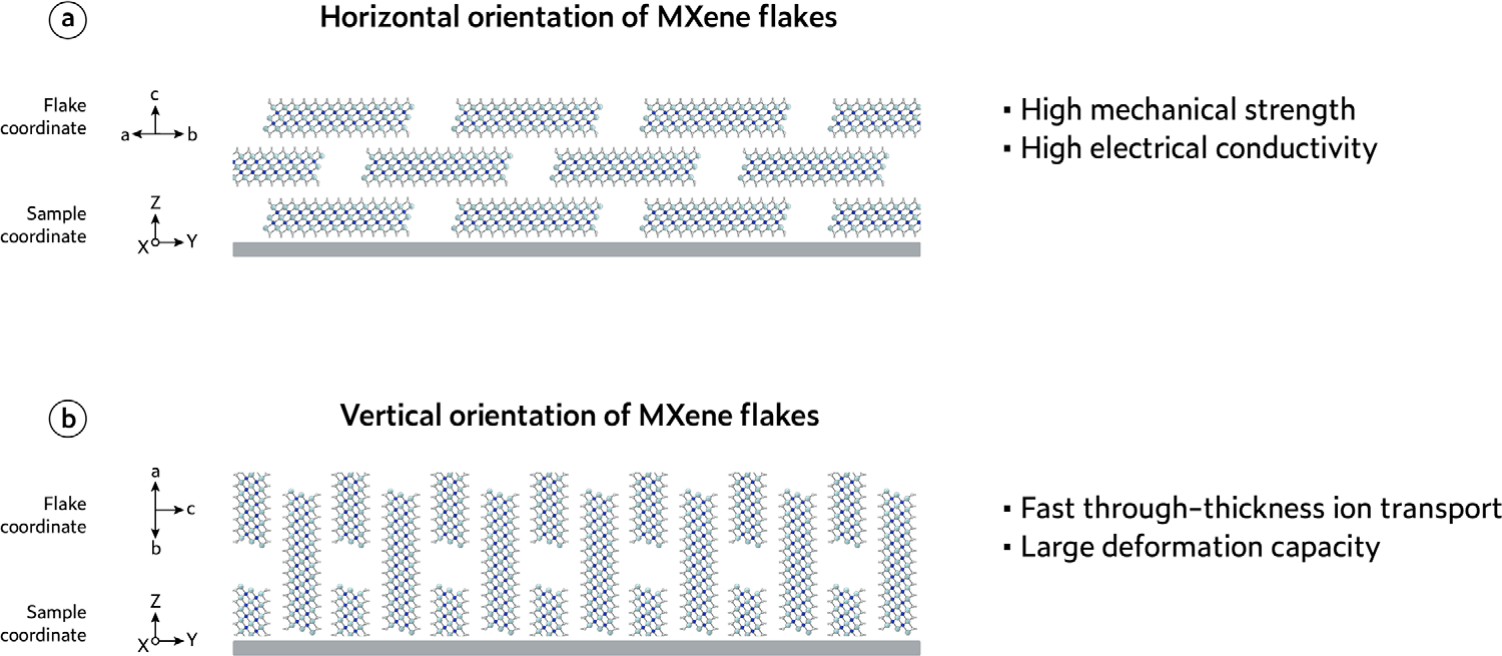
Schematic illustration showing the (a) horizontal and (b) vertical orientations of MXene flakes and their characteristics.

Over the past decade, MXene has garnered widespread attention primarily due to its unique material properties, including compositional diversity, metallic conductivity, rich surface chemistry, high hydrophilicity, large specific surface area, remarkable mechanical strength, and outstanding flexibility. These attributes render them highly promising for a wide range of applications across multiple fields. Meanwhile, the manifestation of these properties in macroscopic MXene assemblies, such as films, membranes, gels, and fibers, is strongly influenced by the orientation of individual flakes. As MXene is a typical 2D layered nanomaterial, its performance can be significantly affected by two common orientation modes (Figure 4), i.e., horizontal orientation (MXene flakes are parallel to the film substrate or fiber axis), and vertical orientation (MXene flakes are perpendicular to the film substrate or fiber axis). Compared to MXene structures with poorly oriented flakes, rational orientation engineering toward these two preferred orientations can overcome such limitations and enable targeted optimization of the functional properties of MXene assemblies, which is discussed in detail below.

Electrical conductivity. MXene has excellent metallic electrical conductivity, which gives it significant advantages for replacing metal contacts in energy and electronic fields. To date, the highest conductivity reported for MXene is 35 000 S cm^−1^ (Ti*_x_* C*_y_* N*_x‐y_*_‐1_T*_z_* MXene films with optimized N content) [27]. However, it is important to note that while MXene exhibits excellent intraflake electron transport, its interflake electron transport capability is relatively weak [28]. Therefore, disordered stacking leads to interflake voids and discontinuous interfaces, which significantly increase the interfacial resistance and hinder efficient charge transport across the MXene assembly. When the MXene flakes exhibit a uniform and consistent horizontal orientation, the interflake contact becomes tighter, thereby providing better electron conduction within the film, and as a result, such highly oriented MXene assembly can achieve significantly superior conductivity compared to its non‐oriented form. By tailoring the orientation of MXene flakes to optimize electrical conductivity, their functional applications in electromagnetic interference (EMI) shielding, energy storage electrodes, and conductors for flexible electronics can be significantly enhanced.

Ion/molecule transport. MXene possesses abundant surface terminations that can interact with various ions and molecules, thereby enabling selective reaction, sensing, and sieving functionalities [29]. Crucially, these functions rely on ion/molecule transport through nanoconfined interflake channels, whose accessibility and efficiency are strongly governed by the orientation of MXene flakes in the assembly. With random orientation, the disordered stacking of MXene flakes creates nonuniform interflake gaps and microcracks, resulting in discontinuous and tortuous ion/molecule transport pathways. This structural disorder leads to inefficient channel accessibility, low utilization of active surfaces, and limited interaction with the transported species. In the case of horizontally oriented flakes, ions or molecules diffuse laterally between layers. This in‐plane transport increases tortuosity and path length, which can impede high‐throughput conduction, but the extended interfacial contact along the basal planes provides additional opportunities for strong and selective interactions with transporting species. In contrast, vertically oriented MXene flakes form well‐defined through‐thickness channels that enable direct and efficient ion/molecule transport across the layers, greatly reducing diffusion distances and enhancing conduction kinetics [30]. By optimizing flake orientation to match targeted ion/molecule transport modes, MXene can achieve enhanced performance in applications such as sensing, electrochemical energy storage, and ion/molecule sieving systems.

Mechanical properties. A MXene monolayer has excellent mechanical strength (Young's modulus of ∼500 GPa) [31], but the mechanical properties of its assemblies are strongly dependent on the orientation of the constituent flakes. In randomly oriented MXene structures, the flakes are misaligned, which leads to weak interflake interactions, poor stress transfer, and interflake slippage, which consequently cause low tensile strength and poor mechanical robustness. In contrast, horizontally oriented MXene flakes form densely packed architectures with uniform face‐to‐face van der Waals interactions and limited interflake slippage, which enables high utilization of the intrinsic mechanical strength of individual flakes and efficient stress transfer along the in‐plane direction, thus significantly improving tensile strength and flexibility [32]. When vertically oriented, MXene flakes rely primarily on weak interflake interactions to sustain external stress, resulting in limited tensile strength. However, by effectively utilizing the interflake spacing, such assemblies can offer potential advantages in structural flexibility and mechanical adaptability toward stretching, compressing, or bending. Optimizing MXene flake orientation for desired mechanical properties enables their application in flexible electronics and advanced devices with adaptive mechanical properties.

Optical transmittance. Owing to their atomic‐scale thickness, MXene possesses intrinsically high optical transmittance, reported as ∼97% for a single layer of Ti_3_C_2_T*_x_* [33]. Nevertheless, the macroscopic transparency of MXene assemblies is not solely determined by their intrinsic absorption but also by the degree of flake orientation. Thin films with horizontally oriented MXene flakes typically exhibit good optical transmittance due to the formation of smooth and continuous surfaces that minimize light scattering [34]. In contrast, randomly stacked MXene flakes introduce interfacial roughness and discontinuities, leading to increased scattering and reduced transparency. Moreover, maintaining a high degree of horizontal orientation over large areas improves the optical uniformity of MXene films, preventing spatial variations in transmittance caused by structural inhomogeneity. These advantages make orientation control a key strategy for fabricating high‐performance transparent MXene thin films, which can serve as transparent conductive electrodes for applications such as solar cells, electrochromic devices, and organic light‐emitting diodes [35].

### 2.3 Characterization of MXene Orientation

As the orientation of MXene flakes plays a critical role in determining their functional properties, accurately characterizing their orientation, either relative to the substrate or among neighboring flakes, is essential for understanding and optimizing MXene‐based architectures. In this section, we introduce the key techniques used to characterize MXene orientation.

X‐ray techniques. X‐ray techniques can be used to analyze the interaction between X‐rays and the periodic atomic arrangement in MXene, which offers high precision, is nondestructive, and allows rapid acquisition to the orientation information [36]. For MXene structures with high orientation, despite the random in‐plane rotational distribution of individual flakes, a strong (002) peak is often observed in conventional *θ*‐2*θ* X‐ray diffraction (XRD) measurements. While the position of the (002) diffraction peak reflects the interflake spacing of MXene, the intensity and sharpness of the (002) peak, quantified by its full width at half‐maximum (FWHM), also offer insights into the flake orientation of MXene assemblies. A strong and narrow (002) peak typically indicates a high level of horizontal orientation, while a broader or weaker peak suggests a relatively disordered stacking pattern.

Using 2D detectors can intuitively reveal the orientation of MXene structures through the analysis of Debye‐Scherrer rings [37]. Specifically, 2D wide‐angle X‐ray scattering (WAXS) is highly suitable for the orientation characterization of MXene assemblies. While conventional transmission‐mode WAXS can effectively reveal the bulk structural features of freestanding structures, WAXS with the grazing‐incidence (GI) mode (GIWAXS) [38], which employ extremely small incident angles, are more surface‐sensitive and thus particularly suitable for probing ultrathin or substrate‐supported MXene structures. Analyzing the achieved Debye–Scherrer rings enables accurate evaluation of flake orientation. In such patterns, a uniform ring‐like signal indicates random orientation and disordered stacking of MXene flakes, whereas highly oriented MXene structures give rise to diffraction arcs or even discrete spots.

Building upon this, the azimuthal intensity profile of the (002) reflection can be extracted to calculate the orientation distribution function (ODF), which describes the probability density of MXene flakes oriented at specific azimuthal angles. From this ODF, statistical quantities such as Herman's orientation factor can be derived to provide a more quantitative assessment of the degree of orientation [39]. Similarly, small‐angle X‐ray scattering (SAXS) can also be employed for orientation analysis. Although it typically does not produce classic Debye–Scherrer rings, the anisotropic intensity distribution observed in the elliptical scattering pattern can be analyzed to determine Herman's orientation factor. The formula for Herman's orientation factor (*f*) is shown below:

(1) $$f=\frac{3\langle \cos^{2}\phi \rangle -1}{2}$$

where *ϕ* refers to the azimuthal angle of the (002) peak of MXene, and 〈cos^2^ *ϕ*〉 is the average value of the square of the cosine of the azimuthal angle. 〈cos^2^ *ϕ*〉 can be calculated using the intensity at an azimuthal angle (*I(ϕ)*):

(2) $$\langle \cos^{2}\phi \rangle =\frac{\int_{0}^{\pi /2}I\left(\phi \right)\sin\phi \cos^{2}\phi d\phi }{\int_{0}^{\pi /2}I\left(\phi \right)\sin\phi d\phi }$$

When used to quantitatively assess the orientation of MXene flakes, a Herman's orientation factor of 1 indicates that the flakes are perfectly oriented parallel to the reference direction (typically the substrate), reflecting a highly horizontal orientation. A value approaching 0 corresponds to randomly oriented flakes, while a value of −0.5 implies perfect vertical orientation [40].

**FIGURE 5:**
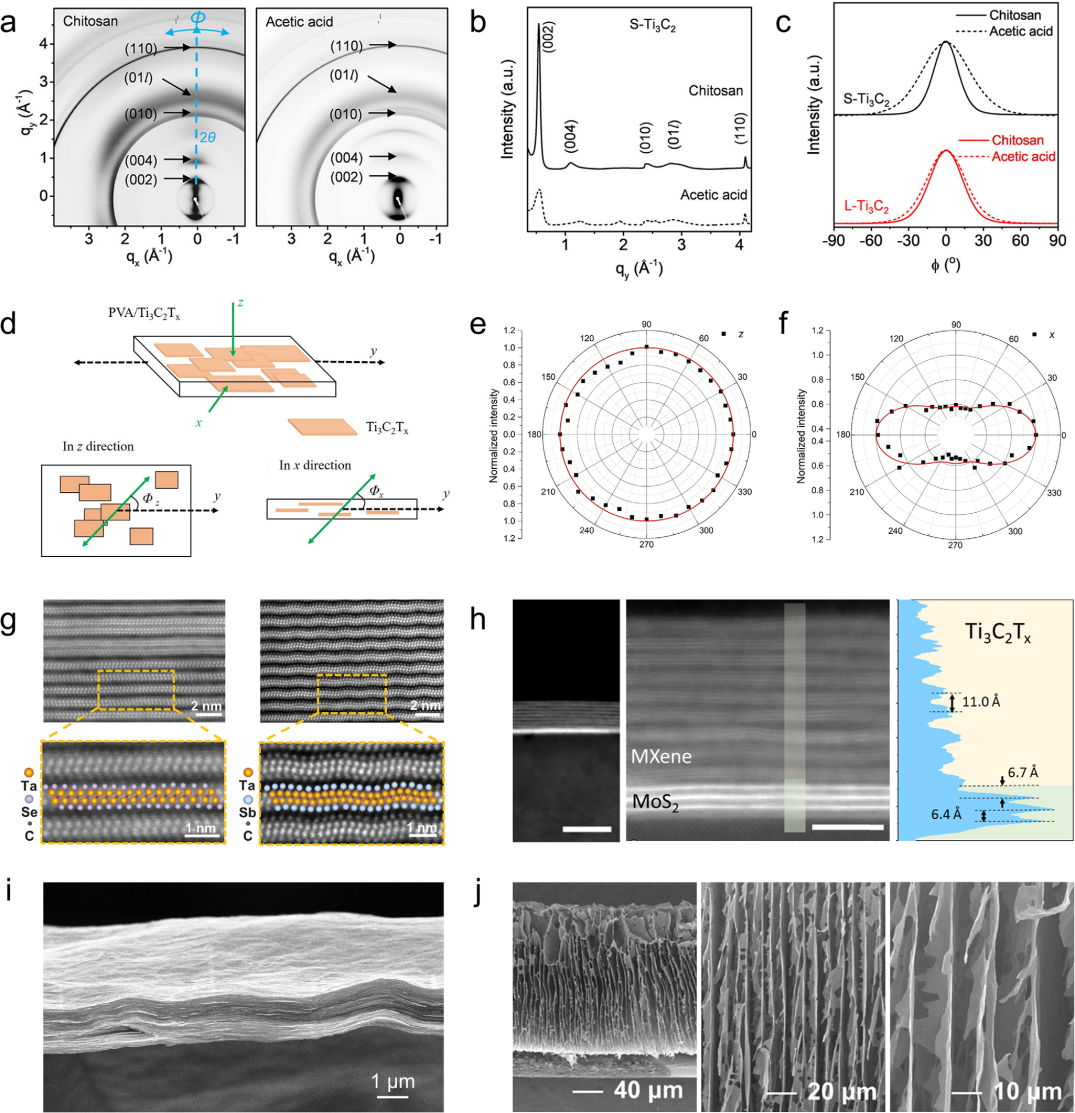
Characterization of MXene flake orientation. (a) SAXS/WAXS patterns of S‐Ti_3_C_2_ fibers prepared with different bathing environments. (b) Radial intensity profile integrated over ±5° along the equatorial direction and normalized to the (110) peak. (c) Azimuthal intensity profile of the (002) scattering signal plotted along the *ϕ* direction within ±90°. Reproduced under the terms of the CC‐BY 4.0 license [41]. Copyright 2020, American Chemical Society. (d) Schematic illustration showing the experimental setup for testing the MXene flake orientation within a PVA/Ti_3_C_2_T*_x_* composite using polarized Raman spectroscopy. Polar plots showing the angular dependence of the normalized A_1g_ Raman intensity of Ti_3_C_2_T*_x_* under VV polarization, with the excitation laser incident along (e) the *z*‐axis and (f) the *x*‐axis. Reproduced with permission [42]. Copyright 2023, The Authors. Published by Elsevier. (g) Cross‐sectional STEM images of Ta_2_CSe*_x_* MXene (right) and Ta_2_CSb*_x_* (right) MXene. Reproduced with permission [43]. Copyright 2023, The American Association for the Advancement of Science. (h) Cross‐sectional STEM images showing the Ti_3_C_2_T*_x_* MXene film on MoS_2_ substrate at different magnifications and the corresponding intensity profile. Reproduced under the terms of the CC‐BY 4.0 license [44]. Copyright 2023, The Authors, published by American Chemical Society. (i) SEM image showing a Ti_3_C_2_T*_x_* MXene fiber with horizontally oriented flakes. Reproduced with permission [45]. Copyright 2024, Wiley. (j) SEM image showing a Ti_3_C_2_T*_x_* MXene film with vertically oriented flakes. Reproduced with permission [46]. Copyright 2024, Wiley.

For example, small‐flake Ti_3_C_2_T*_x_* MXene (S‐Ti_3_C_2_) fibers prepared using chitosan or acetic acid bathing both exhibit highly oriented structures, as shown in the SAXS/WAXS data (Figure 5a) [41]. Integrating along the equatorial direction reveals that the chitosan bathing‐derived fiber possesses a narrower FWHM for the (002) peak compared to the acetic acid‐based one (Figure 5b). Unlike large‐flake Ti_3_C_2_T*_x_* (L‐Ti_3_C_2_) fibers, S‐Ti_3_C_2_ fibers spun in different baths exhibit significant differences in the azimuthal intensity distribution of the (002) peak (Figure 5c). The Herman's orientation factors calculated for S‐Ti_3_C_2_ fibers in chitosan or acetic acid bathing are 0.83 and 0.58, respectively, indicating that the chitosan‐based fiber exhibits a significantly higher orientation along the fiber axis.

Polarized Raman spectroscopy. Raman spectroscopy is a fast and nondestructive technique that provides insights into the vibrational modes, crystal structure, and chemical composition of materials. It can be used to characterize MXene with diverse chemistries and structures, and even MXene species with similar compositions but different layer thicknesses often exhibit markedly different Raman spectra. When polarized light is used as the excitation source, the Raman response of MXene depends on both the symmetry of the vibrational mode and the crystal orientation. This feature makes polarized Raman spectroscopy suitable for evaluating the flake orientation in MXene structures [47]. The A_1g_ vibrational mode of MXene, which involves out‐of‐plane atomic displacements, is highly sensitive to polarization aligned with the flake normal [48]. When the laser is incident parallel to the flake plane and the sample is scanned azimuthally, it exhibits pronounced angle‐dependent intensity variations.

Analogous to orientation analysis using WAXS, the angular intensity distribution of such modes can be used to calculate the Herman's orientation factor, thereby enabling quantitative analysis of MXene flake orientation [49]. It should be noted that polarized Raman spectroscopy is especially suitable for analyzing MXene flake orientation in polymer composites with a low MXene content (e.g., <10%). In such cases, MXene flakes are often sparsely distributed with inconsistent interflake spacing and limited long‐range stacking, which significantly weakens the scattering signals in WAXS measurements. In contrast, polarized Raman spectroscopy does not rely on diffraction‐based signals, making it a more reliable and robust method for orientation analysis under these conditions.

As shown in Figure 5d, polarized Raman spectroscopy was employed to characterize the orientation of MXene flakes within the MXene–poly(vinyl alcohol) (PVA) composite [42]. When the Raman excitation is incident perpendicular to the basal plane of MXene flakes, the A_1g_ mode exhibits isotropic responses in polarized Raman spectroscopy, as evidenced by the nearly uniform intensity across all azimuthal angles (Figure 5e). In contrast, when the excitation light is incident parallel to the basal plane of the MXene flakes, a pronounced variation in azimuthal Raman intensity is observed, from which a Herman's orientation factor of 0.28 can be calculated (Figure 5f).

Transmission electron microscopy (TEM). TEM uses a high‐energy electron beam to penetrate a sample and produce high‐resolution images. For a single MXene flake, TEM enables direct atomic‐scale imaging, allowing analysis of crystal structure, lattice fringes, and defects, while the coupled selected‐area electron diffraction (SAED) technique provides localized crystallographic information. Precisely cutting MXene samples with focused ion beam (FIB) technology to prepare ultrathin slices on the cross‐section and then characterizing them using TEM can reveal high‐resolution information on flake orientation. This approach has been especially used to check the structural configuration of MXene regarding surface termination. For example, cross‐sectional TEM images of the accordion‐like Ta_2_C MXene with different terminations are shown in Figure 5g, where the parallel MXene lattices also indicate the horizontal orientation of the MXene flakes within the accordion [43]. Moreover, FIB‐assisted cross‐sectional TEM can be used to investigate the MXene flakes in thin films, providing direct insights into their orientation behaviors. Figure 5h presents the TEM image of the cross‐section of a Ti_3_C_2_T*_x_* MXene film [44]. The highly ordered and parallel MXene flakes clearly demonstrate the horizontal MXene orientation within the structure. Conducting SAED on the cross‐section of an oriented MXene structure can also be used to quantitatively evaluate flake orientation by analyzing the diffraction spots, i.e., the more concentrated the spots, the higher the degree of orientation. However, its application is limited due to the complexity of cross‐sectional sample preparation, and this function is well covered by X‐ray techniques that have been mentioned above.

Scanning electron microscopy (SEM). SEM provides high‐resolution images of surface morphology by scanning the sample with a focused electron beam. While SEM does not yield direct crystallographic information, it is widely used to visualize the overall arrangement and packing of MXene flakes within films, fibers, or other assemblies. Highly compact and uniform stacking generally indicates a high degree of flake orientation, whereas disordered or loosely packed structures are typically associated with poor orientation. By referencing the substrate as an orientation baseline, we can distinguish between the vertical and horizontal orientation of the MXene flakes. For example, Figure 5i,j illustrates MXene flakes with horizontal and vertical orientations, respectively [45, 46]. Cryo‐SEM offers the potential to capture transient intermediate states during orientation engineering by freezing the structure in its native wet form [50], yet its application remains limited in current MXene orientation research. It is worth noting that although electron backscatter diffraction is a powerful SEM‐based technique for crystallographic orientation characterization, its application in MXene orientation analysis has been rarely reported, possibly due to insufficient backscattered electron signals caused by the combined effects of lightweight constituents, limited flake size, and difficulties in achieving the required mirror‐like surface smoothness.

The techniques described above are the most commonly used methods for characterizing the orientation of MXene flakes. Depending on the specific form and structure of the MXene sample, suitable methods can be selected to probe the orientation information. Among these, WAXS has become the most widely adopted approach, owing to its ability to provide Debye–Scherrer ring signals and enable quantitative analysis of orientation via Herman's orientation factor. Collectively, these techniques offer a comprehensive toolkit for characterizing the orientation of MXene flakes across multiple length scales and structural dimensions.

## 3 Strategies for Orientation Engineering of MXene Flakes

In this review, methods such as surface tension‐ and capillary force‐driven orientation are categorized as self‐assembly strategies, as they rely on intrinsic interfacial interactions and solvent dynamics without the application of external fields or mechanical forces. In contrast, orientation techniques involving external stimuli or engineered physical constraints are discussed under externally assisted methods.

### 3.1 Self‐Assembly Methods

**FIGURE 6:**
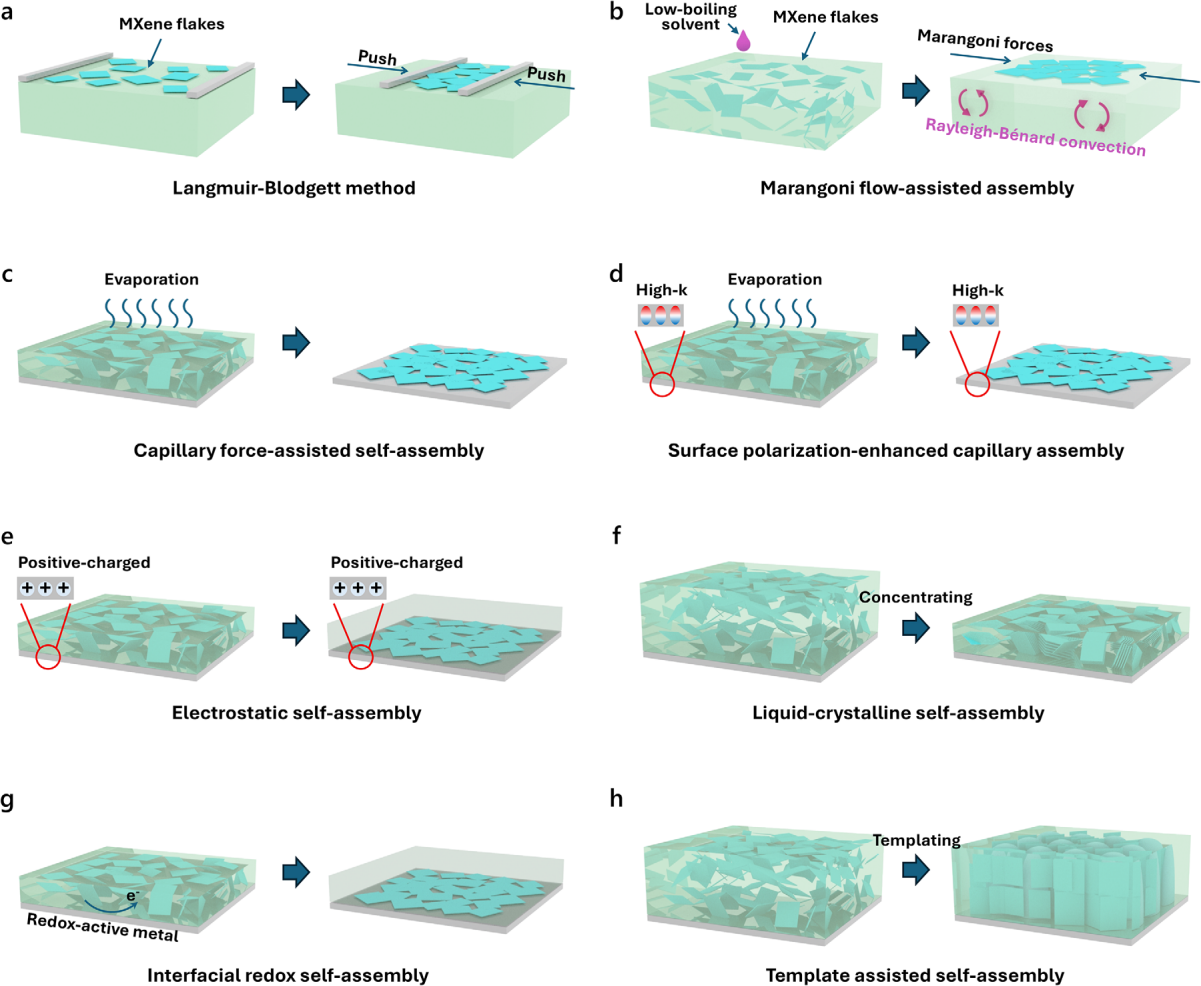
Self‐assembly strategies for orientation engineering of MXene flakes. Schematic illustration showing the process of (a) Langmuir–Blodgett method, (b) Marangoni flow‐assisted assembly, (c) typical and (d) surface polarization‐enhanced capillary force‐assisted self‐assembly, (e) electrostatic self‐assembly, (f) liquid‐crystalline self‐assembly, (g) interfacial redox self‐assembly, and (h) template‐assisted self‐assembly.

Unlike many other 2D materials (such as graphene), MXene is naturally hydrophilic and easily dispersed in aqueous solutions, which gives it unique advantages in solution processing and subsequent self‐assembly. Self‐assembly methods provide a spontaneous and energy‐efficient pathway for inducing orientation in MXene‐based structures. Compared to external‐field‐driven methods, self‐assembly relies on its own physicochemical properties of MXene, such as surface terminations, negative charges, and 2D geometries, to achieve directional organization under controlled conditions. In the following content, several distinct self‐assembly strategies exploiting different driving forces for MXene orientation engineering are discussed (Figure 6).

Surface tension represents a key physicochemical mechanism underlying the interfacial self‐assembly of MXene flakes into oriented structures. Langmuir–Blodgett (LB) assembly is a typical approach based on it (Figure 6a) [51]. In this method, MXene flakes are first spread at the air–water interface, where they spontaneously orient along the liquid surface due to surface tension and interfacial interactions. Through controlled compression of the barrier and subsequent vertical lifting of the substrate, an oriented thin film of horizontally oriented MXene flakes can be transferred onto solid substrates. This approach enables precise control over film thickness and orientation, making it attractive for applications requiring uniform anisotropic properties.

Another representative example is Marangoni‐driven assembly, which is based on the Marangoni effect, i.e., liquid flow induced by surface tension gradients, to realize interfacial self‐assembly of oriented MXene structures (Figure 6b) [52]. Typically, a droplet of low‐tension solvent containing dispersed MXene flakes is deposited onto another high‐tension liquid surface. The surface tension difference drives the MXene flakes to migrate toward regions of higher surface tension, leading to the formation of uniform monolayered or multilayered MXene films. This process can also yield horizontally oriented MXene films, while offering a faster fabrication route and eliminating the need for specialized equipment compared to the LB method.

Capillary forces arising during solvent evaporation are another fundamental driving force for inducing the spontaneous orientation of MXene flakes (Figure 6c) [53]. As the solvent gradually evaporates from a dispersion drop or film, the receding liquid meniscus generates lateral capillary flow and confinement effects, driving the flakes to orient parallel to the substrate to minimize surface energy and packing frustration. This mechanism is particularly effective when the evaporation process is slow and uniform, allowing the flakes sufficient time to re‐orient into compact, ordered configurations. Such evaporation‐induced assembly approach enables the fabrication of horizontally oriented MXene films via techniques such as drop‐casting, dip‐coating, and spray‐coating [54], and is particularly appealing due to its simplicity, scalability, and good compatibility with diverse substrates.

Building upon the evaporation‐induced assembly strategy, a surface polarization‐assisted approach was further developed to enhance the formation of horizontally oriented MXene films (Figure 6d) [44]. When MXene films were prepared via evaporation on different substrates, those with a high dielectric constant could form strong interfacial interactions with MXene through a surface polarization phenomenon. This effect facilitated tight adhesion between the MXene flakes and the substrate, enabling the retention of a uniform, horizontally oriented MXene thin film with a sub‐10 nm thickness after subsequent rinsing steps to remove loosely bound flakes. In contrast, substrates with low dielectric constants failed to induce sufficient polarization, resulting in discontinuous, island‐like MXene residues under the same processing conditions.

Utilizing the surface terminal groups of MXene flakes provides an alternative route for their self‐assembly into oriented structures. Electrostatic interfacial assembly is a widely utilized strategy for directing the orientation of MXene flakes, which exploits the surface charge characteristics of MXene (Figure 6e) [55]. Due to the negatively charged functional groups on MXene surfaces, electrostatic interactions with oppositely charged substrates, polymers, or interfacial environments can induce horizontal orientation during the assembly process. Such charge‐guided self‐organization enables the formation of well‐ordered MXene films or hybrid structures, and is particularly effective in constructing alternating multilayer (superlattice‐like) architectures with controlled orientation.

Another approach related to surface functional groups is liquid crystalline self‐assembly (Figure 6f) [56]. MXene flakes, due to their high aspect ratio and surface terminations, can interact strongly with polar solvents and other charged species in dispersion, leading to spontaneous formation of lyotropic liquid crystalline phases at relatively high concentrations. Most reported MXene‐based liquid crystalline phases are of the nematic type, characterized by mesoscopic orientational ordering of flakes without positional order. Flake size plays a critical role in the formation of MXene liquid crystal phases: larger flakes can form liquid crystals at relatively low concentrations, while smaller flakes often require much higher concentrations [41]. By employing suitable surfactants and substantially increasing the MXene concentration, a smectic liquid‐crystal phase of MXene can be obtained [57]. Although liquid crystalline self‐assembly alone often falls short of producing macroscopically long‐range oriented MXene structures, it serves as a powerful strategy when combined with auxiliary external forces such as shear to realize the fabrication of highly oriented architectures. Interestingly, under identical shear conditions, nematic and smectic phases respond in fundamentally different ways: the former orients horizontally to the substrate, whereas the latter adopts a vertical orientation [57].

Furthermore, the oxygen‐containing surface terminations of MXene possess a certain degree of oxidizing ability, enabling them to accept electrons from a reductive substrate such as Zn, which promotes the aggregation and oriented stacking of MXene flakes on the reductive substrate (Figure 6g) [58]. During this interfacial redox process, metallic Zn is oxidized to Zn^2+^, and such in situ generated multivalent ions further enhance interflake interactions by providing ionic bonding between adjacent flakes [59].

For the aforementioned self‐assembly mechanisms, employing a specific structural template can further guide the organization of MXene flakes into an ideal manner (Figure 6h). A widely adopted strategy is using ice as the template [60]. In this process, specially designed molds are used to induce directional growth of ice crystals within the MXene solution. The growing ice crystals serve as templates, guiding the assembly of MXene flakes within the gaps between ice crystals. Freeze‐drying is then employed to remove the ice crystals while preserving the MXene structure, where the growth direction of the ice crystals determines the orientation of the MXene flakes in the as‐achieved architecture. Beyond ice templating, hard and soft templates can also guide the oriented self‐assembly of MXene. For example, Song et al. employed a line‐patterned polydimethylsiloxane mold as the template, where the confined geometry of the template promotes vertical orientation of MXene flakes upon solvent evaporation [61]. By incorporating template‐assisted approaches, the self‐assembly of MXene can be guided toward a broader range of oriented structures, thereby offering enhanced control over structural anisotropy.

In summary, diverse self‐assembly strategies developed to achieve oriented MXene architectures have been introduced above. These approaches exploit a variety of driving forces, such as interfacial tension, capillary dynamics, surface charge interactions, and chemical redox potentials, to guide the directional orientation of MXene flakes. Together, they offer a versatile and scalable toolbox for constructing anisotropic architectures, enriching the design flexibility and functional tunability of MXene materials for advanced applications.

### 3.2 External Force‐Assisted Orientation

**FIGURE 7:**
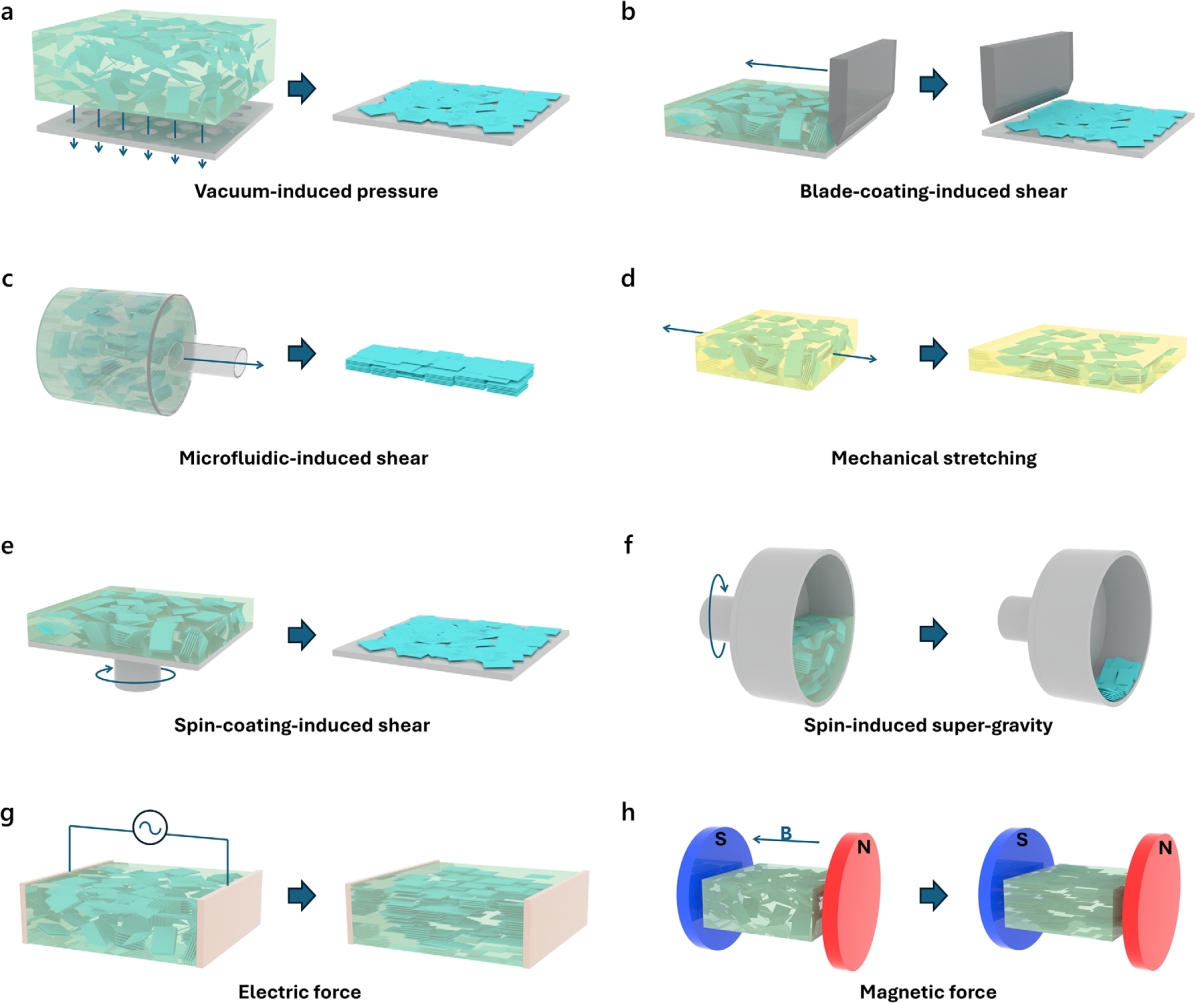
External force‐assisted strategies for orientation engineering of MXene flakes. Schematic illustration showing the external forces of (a) vacuum‐induced pressure, (b) blade‐coating‐induced shear, (c) microfluidic‐induced shear, (d) mechanical stretching, (e) spin‐coating‐induced shear, (f) spin‐induced supergravity, (g) electrical force, and (h) magnetic force.

In addition to spontaneous self‐assembly, external fields and forces can serve as powerful tools to control the orientation of MXene flakes during structure formation. This responsiveness originates from their intrinsic high aspect ratio, surface charge, and flexibility, which allow the flakes to re‐orient under flow, shear, electric, or magnetic fields. Compared to self‐assembly, field‐assisted methods offer improved controllability and uniformity, which is critical for optimizing the orientation‐dependent properties of MXene‐based materials. Various external force‐assisted orientation strategies are discussed in detail as follows (Figure 7).

Vacuum‐assisted filtration is a widely used method for fabricating oriented MXene membranes due to its simplicity in generating well‐oriented layered structures (Figure 7a) [62]. During the filtration process, the solvent is directionally removed through the membrane under vacuum. With the effect of geometrical confinement and the solvent flow, the MXene flakes are forced to stack parallel to the surface of the filter membrane. The thickness of the MXene membranes can be easily tuned by varying the amount of MXene in the filtration dispersion. In addition, the degree of orientation can be optimized by adjusting parameters such as MXene concentration, flake size, filtration rate, filter membrane type, and postfiltration drying conditions. In addition, some polymer additives can help stabilize the MXene dispersion and thus promote the formation of densely packed, ordered lamellar MXene structures [63]. However, the overall orientation of the resulting MXene membranes tends not to be particularly high due to the limited controllability of the filtration process. Despite this limitation, vacuum‐assisted filtration is still widely used for MXene structures owing to its low cost and ease of operation, which makes it suitable for applications where orientation or surface flatness is not very critical. Moreover, it is often adopted as a benchmark method for comparison when evaluating newly developed MXene orientation engineering strategies.

Regarding the use of shear forces, blade coating serves as a typical example for engineering the orientation of MXene flakes (Figure 7b) [64]. Typically, for blade coating, MXene dispersions need to be concentrated to a high enough level to form a liquid crystalline phase, in which the MXene flakes exhibit orientational responsiveness to external shear forces. During the blade coating process, the shear force generated between the moving blade and the substrate induces directional flow within the liquid crystalline domains, effectively orienting the MXene flakes along the coating direction. Beyond conventional blade coating, several derivative techniques, such as slot‐die coating, have been reported for preparing MXene films with highly horizontal orientation [65]. These derivative methods provide enhanced control over film thickness, shear uniformity, and process reproducibility, which are crucial for producing large‐area MXene films with consistent orientation. Due to the compatibility with roll‐to‐roll processing, these blade coating methods offer a promising route toward the large‐scale, continuous production of highly oriented MXene films.

Similarly, shear forces have a significant impact on the fabrication of MXene fibers, and the MXene spinning dope for the spinning process also relies on a liquid crystalline phase (Figure 7c) [66]. During the microfluidic spinning process of fiber preparation, shear forces primarily originate from the velocity gradient generated between the flowing spinning dope and the nozzle wall, which induces axial orientation of MXene flakes within the confined channel. In fiber spinning, circular nozzles typically yield the resulting MXene fibers with circular cross‐sections. While the flakes are well‐oriented along the fiber axis, their orientation in the radial direction remains random. Li et al. demonstrated that altering the nozzle geometry from circular to flat can modulate the distribution of shear forces during spinning, thereby promoting more consistent orientation of MXene flakes in the cross‐section [67]. Additionally, designing a contraction–expansion channel can generate a shear gradient during spinning, which can induce a transition in MXene flake orientation from being perpendicular to the cross‐section to being parallel to it [68]. By tailoring the shear forces during MXene fiber fabrication, the orientation of MXene flakes can be tuned both axially and radially, thereby enabling the modulation of key properties such as electrical conductivity, mechanical strength, and ionic transport.

Mechanical drawing is an effective strategy to enhance the orientation of MXene flakes after initial MXene structure formation (Figure 7d) [69]. This process is particularly beneficial for systems in which the initial orientation of MXene flakes is incomplete or disrupted during solidification, such as semisolid or gel‐like MXene‐containing materials. Upon the application of uniaxial tensile stress, the embedded flakes experience anisotropic stress distribution that drives them to rotate, slide, and reorient their basal planes parallel to the stretching direction, thereby minimizing internal stress concentrations and improving load transfer efficiency [70]. Such strategy has been demonstrated to effectively improve the orientation of MXene flakes within fiber structures [71]. In addition, it is worth noting that the stress‐induced reorientation of MXene also modulates its electrical conductivity, which enables the design of MXene‐based strain‐sensing materials and devices with tunable electromechanical responses [72].

Another widely used method for preparing oriented MXene structures is spin‐coating (Figure 7e). In spin‐coating, the high‐speed rotation induces radial shear flow and centrifugal spreading, thus making the MXene flakes orient parallel to the substrate [33]. With such strong radial shear flow, spin‐coating typically yields MXene films with stronger orientation compared to vacuum filtration. Spin‐coating can achieve relatively uniform MXene film thickness on small substrates, such as glass plates or chips, and the film thickness can be effectively controlled by varying the MXene dispersion concentration and the spinning speed. However, when applied to large areas, thickness variations can occur between the center and the edges, making it challenging to obtain uniformly oriented MXene structures over a large area. Moreover, it should be noted that spin‐coating requires significantly higher MXene concentrations, or else it will fail to produce continuous films, and most of the MXene dispersion is lost during spinning and only a small fraction remains on the substrate to form the film, resulting in significant material waste.

High‐gravity fields, typically generated by centrifugal force, have also been explored as an effective strategy to induce the orientation of MXene flakes (Figure 7f). Under centrifugal conditions, supergravity fields exceeding a thousand times Earth's gravity can be readily achieved. Such a process enables the rapid and directional sedimentation of MXene flakes suspended in a liquid or gel matrix, achieving efficient orientation engineering within a short processing time [73]. Although both spin‐coating and supergravity‐assisted methods rely on centrifugal forces, the substrate where the material is collected differs significantly. In spin‐coating, the film forms directly on the rotating flat substrate, whereas in supergravity‐induced deposition, the MXene flakes are driven radially outward and accumulate along the inner wall of the centrifuge container, forming a film perpendicular to the rotation axis. Beyond film fabrication, this supergravity‐assisted method has also been reported to be highly effective for regulating the dispersion and orientation of MXene flakes within polymer composites [74].

Apart from mechanically induced orientation strategies, external physical fields such as electric fields have also proven effective for manipulating the orientation of MXene flakes (Figure 7g). Due to their inherent surface charge and high conductivity, MXene flakes are highly responsive to electric fields [75]. A direct current field generally induces electrophoretic migration and deposition of MXene flakes onto the electrode surface. By contrast, an alternating current (AC) field generates torque of the MXene flakes, allowing their basal planes to reorient along the field lines. By adjusting the direction of the applied field, programmable control over flake orientation can be achieved [76]. It is worth noting that the concentration of the MXene suspension should be sufficiently high in AC conditions, or else electrophoretic deposition may dominate the process under the applied electric field. Moreover, by patterning the direction and configuration of the applied field, the electric‐field‐assisted strategy enables precise control over the orientation of MXene flakes within complex geometries [77]. This capability enables the programmable design of structure‐function relationships by controlling the orientation of MXene flakes across spatially complex or architecturally diverse configurations.

A magnetic‐field‐assisted method has also emerged as a viable strategy for controlling the orientation of MXene flakes (Figure 7h). Although pristine MXene flakes are generally nonmagnetic, the magnetic responsiveness of MXene flakes can be achieved by incorporating magnetic nanoparticles (e.g., Fe_3_O_4_) onto the MXene surfaces [78]. Under an external magnetic field, magnetically decorated MXene flakes tend to align their basal planes parallel to the field direction due to field‐induced torque acting on their anisotropic structure. Compared to static magnetic fields, rotating magnetic fields can provide continuous torque, enabling more effective and uniform orientation of magnetically functionalized 2D flakes [79]. Such approach is well‐suited for MXene‐based polymer composites, as it enables non‐invasive, spatially uniform control over flake orientation within viscous or crosslinkable matrices, and the oriented structure can be further fixed via in situ polymerization or gelation [80].

In summary, external physical fields such as shear fields, electric fields, and magnetic fields offer versatile strategies to regulate the orientation of MXene flakes. The application of external fields, in conjunction with the intrinsic self‐assembly behavior of MXene, enables effective fabrication of highly oriented MXene structures.

As shown in Table 1, the major orientation‐engineering strategies are concisely summarized, together with their key advantages and main limitations. In general, self‐assembly routes (surface tension, capillary force, electrostatic interaction, interfacial redox, and template‐assisted assembly) operate under mild conditions and can readily yield horizontally oriented MXene architectures with good uniformity, making them particularly attractive for ultrathin films and multilayer heterostructures. Their limitations typically stem from the sensitivity to environmental parameters (such as temperature, humidity, and wind) and chemical conditions (such as dispersion chemistry, interfacial cleanliness, and reaction kinetics), which may lead to variability, transfer‐induced defects, and challenges in scaling up. By contrast, external‐force‐assisted approaches (vacuum pressure, shear force, supergravity, and electric/magnetic fields) offer improved controllability and can generally enhance the scalability and the manufacturability of the oriented MXene structures. However, these external‐force‐assisted approaches rely on specialized equipment and a narrow processing window, which typically requires extensive process optimization for large‐scale manufacturing. In device‐relevant scenarios, strategy selection should be guided by the target film thickness, orientation mode, and manufacturability requirements. For example, when large‐area, highly oriented freestanding films or fibers are required, shear force‐assisted strategy (such as blade coating or wet spinning) is often a preferred route due to its scalability. Conversely, when on‐chip, horizontally oriented MXene monolayer films are desired, surface‐tension‐driven interfacial self‐assembly followed by transfer onto the target substrate can be a good solution.

**TABLE 1:** Comparison of major MXene orientation‐engineering strategies.

Orientation engineering strategy		Orientation mode	Key advantages	Limitations
Self‐assembly	Surface tension	Horizontal	Simple setup, ultrathin films, interfacial transfer	Limited thickness, sensitive to contaminants
	Capillary force	Horizontal	Low‐cost, good compatibility, tunable thickness	Sensitive to environment, drying‐induced defects
	Electrostatic interaction	Horizontal	Precise thickness control, programmable structure, good uniformity	Time‐consuming, hard to reach large‐area thick films
	Interfacial redox	Horizontal	Enhanced interfacial property, potential for functional composites	Chemistry‐dependent, risk of MXene oxidation, kinetically sensitive
	Template assisted	Horizontal/vertical	Dimensional flexibility, high thickness	Additional steps and cost, potential defects from demolding
External force	Vacuum pressure	Horizontal	Simple operation, wide thickness range,	Area limited by filtration setup, time‐consuming
	Shear force	Horizontal/vertical	Fast operation, scalability, controllable thickness	Sensitive to ink rheology, drying‐induced defects
	Supergravity	Horizontal	Fast operation, controllable via g‐force/time	Nonuniformity on edges, limited sample area
	Electric force	Horizontal/vertical	Rapid and controllable, compatible with specific device architectures	Requires electrodes, sensitive to MXene concentration
	Magnetic force	Horizontal/Vertical	Rapid and controllable, non‐contact process	Requires magnetic functionalization, requires strong fields (equipment)

## 4 Advances of MXene Orientation Engineering

Through the orientation engineering of MXene flakes, MXene‐based materials have demonstrated remarkable potential in various applications, including energy, electronics, and the environment. Across these applications, the desired MXene orientation is largely dictated by where the key transport bottleneck resides. Horizontal orientation is often favored when in‐plane charge transport and film uniformity are most critical, whereas vertical orientation is important when through‐thickness ion/mass transport limits the device. Rather than treating orientation as a descriptive label, the degree of orientation can be quantified (e.g., by Herman's orientation factor) and optimized to enhance the corresponding directional transport processes that govern MXene device performance. Moreover, realizing the intended performance often requires not only generating a desired orientation, but also preserving it throughout device integration and subsequent operation. In the following, a systematic overview of recent advances in different application areas is provided, highlighting how orientation control of MXene flakes contributes to enhanced MXene device performance.

### 4.1 Energy Storage Devices

MXene has garnered significant attention in the field of energy storage due to its high electrical conductivity, tunable surface chemistry, and layered architecture, which allows them to accommodate ions [4]. In such applications, ranging from supercapacitors to rechargeable batteries, the orientation of MXene flakes serves as a critical component in determining ion transport dynamics, electrode kinetics, and long‐term structural stability. In this context, vertical orientation is important for improving ion transport across the electrode thickness, while horizontal orientation can optimize in‐plane electron transport and structural integrity.

**FIGURE 8:**
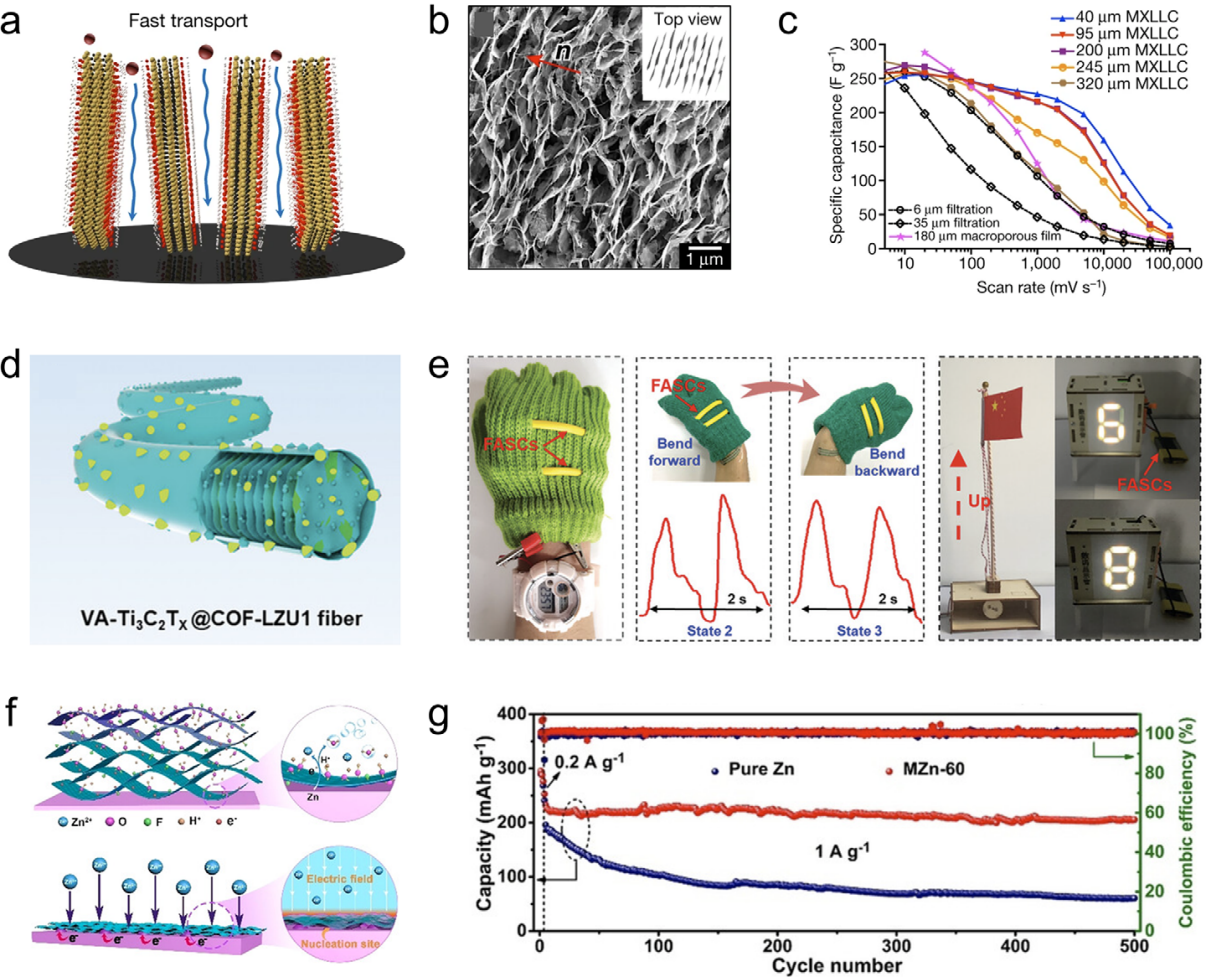
Orientation engineering of MXene flakes for energy storage devices. (a) Schematic illustration showing fast ion transport in vertically oriented MXene film. (b) Top‐view SEM image showing the vertically oriented MXene flakes in the MXene film. (c) Rate performance of different MXene samples evaluated at various scan rates. Reproduced with permission [57]. Copyright 2018, Macmillan Publishers Ltd., part of Springer Nature. (d) Schematic illustration showing a vertically oriented MXene–COF fiber. VA refers to vertical‐aligned. (e) Applications of fiber‐shaped supercapacitors based on such vertical MXene–COF fiber in powering different electronics. Reproduced with permission [81]. Copyright 2023, Wiley‐VCH GmbH. (f) Schematic illustration showing the assembly of horizontally oriented MXene film on Zn foil. (g) Cycling stability of Zn/MnO_2_ batteries using bare Zn anode and Zn/MXene anode (MZn, 60 refers to 60 nm thickness of the MXene coating layer). Reproduced with permission [58]. Copyright 2020, Wiley‐VCH GmbH.

Vertically oriented architectures offer shortened ion diffusion paths across the electrode thickness and enable more efficient ion accessibility to interflake sites, which is crucial for maximizing capacity and rate capability in thick or compact electrodes. Xia et al. reported the application of vertically oriented Ti_3_C_2_T*_x_* MXene films for supercapacitor electrodes, which exhibit electrochemical energy storage performance that is nearly independent of film thickness (Figure 8a) [57]. Traditionally, thick films of 2D materials suffer from severe ion transport limitations due to restacking, but this work overcomes that challenge through the formation of MXene lamellar liquid crystal (MXLLC). A nonionic surfactant (C_12_E_6_) that promotes lamellar ordering of the MXene flakes, followed by mechanical shear to endow the MXene orientation into a vertical mode (Figure 8b). Electrochemical characterization shows that these vertically oriented MXene films retain their pseudocapacitive performance and high specific capacitance (over 200 F g^−1^ at up to 2000 mV s^−1^) nearly regardless of thickness (Figure 8c). Later on, Zhu et al. reported the development of a vertically oriented hybrid fiber electrode based on Ti_3_C_2_T*_x_* MXene and a covalent organic framework (COF–LZU1), fabricated via a one‐step microfluidic self‐assembly strategy (Figure 8d) [81]. The resulting MXene–COF fiber exhibits a well‐ordered vertical orientation and abundant open‐pore structure, enabling efficient ion transport and enhanced redox accessibility. The fiber‐shaped asymmetric supercapacitors based on such fiber demonstrate excellent flexibility and practical applicability by powering wearable electronics (LEDs, watches) and enabling pulse signal detection (Figure 8e).

By contrast, horizontally oriented MXene structures provide continuous in‐plane pathways for electron transport and facilitate fast charge propagation along the electrode surface, which are commonly employed in the construction of current collectors and as protective layers for metal anodes in metal‐based batteries. Zhang et al. presented a facile and effective strategy to stabilize Zn anodes for aqueous zinc‐ion batteries by directly self‐assembling a horizontally oriented Ti_3_C_2_T*_x_* MXene layer on the Zn surface (Figure 8f) [58]. This MXene coating layer with horizontal MXene flake orientation offers multiple advantages, such as enhanced electrolyte wettability, lower Zn nucleation energy barrier, more uniformly distributed electric field, and suppressed Zn dendrite formation. As a result, full Zn//MnO_2_ cells using such anodes deliver high reversible capacities over 500 cycles, indicating significant optimization compared to bare Zn anode (Figure 8g).

### 4.2 Energy Harvesting Devices

Energy harvesting, which aims to convert ubiquitous environmental energy into usable electrical power, encompasses a broad range of mechanisms such as piezoelectric, triboelectric, salinity‐gradient, and electromagnetic energy conversion [82]. Across these diverse domains, MXene orientation engineering plays a unique and critical role. In energy‐harvesting devices, the most suitable MXene orientation is mechanism‐dependent. Horizontal orientation is typically desired when in‐plane electrical conduction and mechanical coupling dominate (such as piezoelectric energy harvesting). On the contrary, vertical orientation is essential for membrane‐based systems where through‐thickness ion transport governs the output (such as osmotic energy harvesting).

**FIGURE 9:**
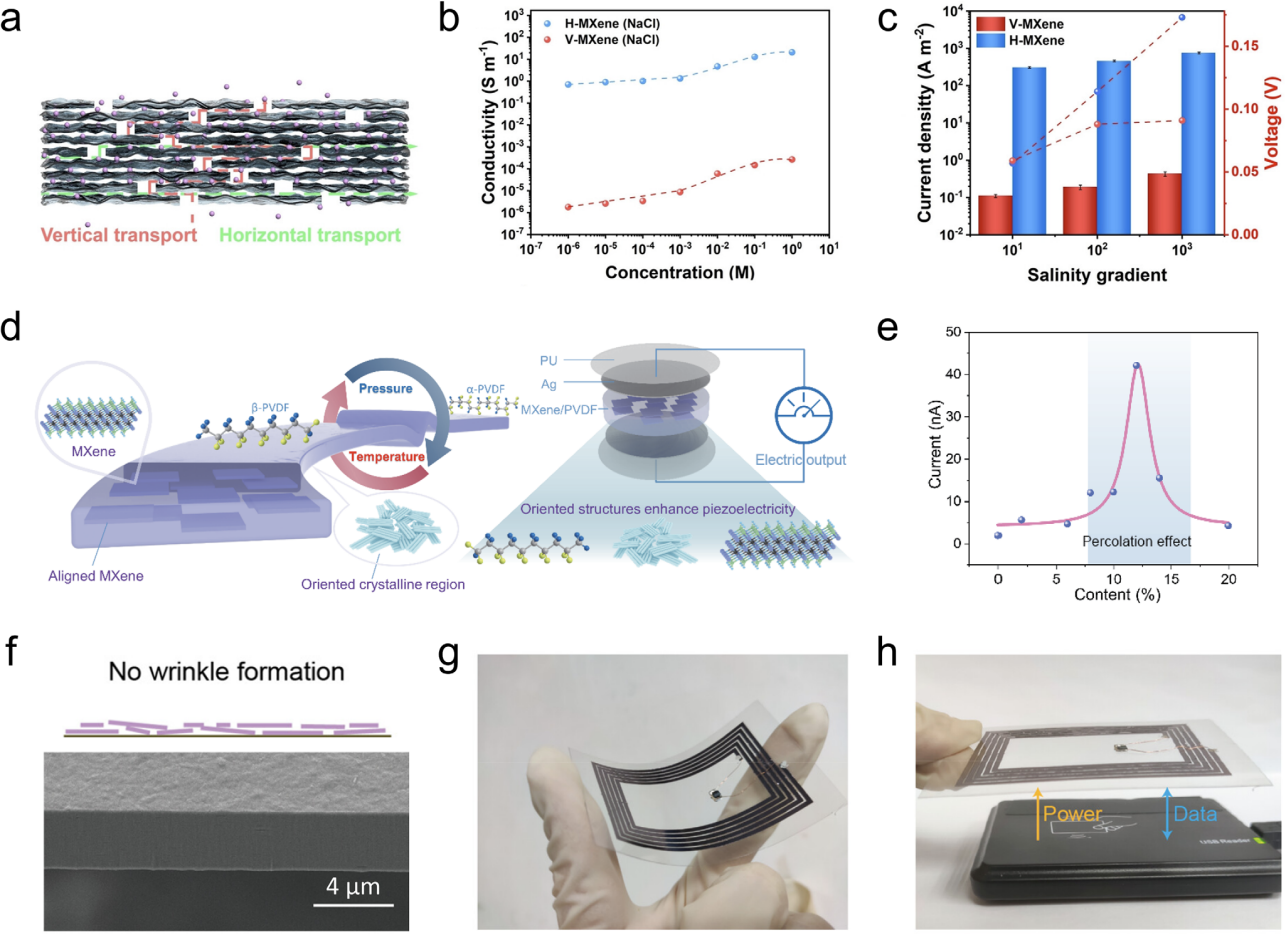
Orientation engineering of MXene flakes for energy harvesting devices. (a) Schematic illustration showing the ion transport along the through‐thickness direction and the in‐plane direction. (b) Ionic conductivity and (c) energy harvesting performance of different oriented MXene structures. Reproduced with permission [84]. Copyright 2024, Wiley‐VCH GmbH. (d) Schematic illustration showing the functionality of oriented MXene flakes in piezoelectric energy harvesting. PU refers to polyurethane. (e) Current output of the piezoelectric device with different MXene content. Reproduced with permission [86]. Copyright 2024, Tsinghua University Press. (f) Schematic illustration and SEM image of MXene films formed under low wet‐film thickness. Photos showing (g) the MXene coil fabricated via such orientation engineering technique and (h) its demonstration in near‐field energy and information transfer. Reproduced with permission [88]. Copyright 2023, American Chemical Society.

For osmotic energy harvesting based on MXene membranes, the layered structure of MXene provides abundant 2D nanofluidic channels for ion transport. In a vertically oriented MXene configuration, these interflake channels are aligned along the ion transport direction, allowing ions to pass directly through the gaps between adjacent MXene flakes, which can significantly shorten the ion diffusion path and reduce internal resistance, thereby facilitating fast transmembrane ion migration and enhancing energy harvesting performance of the osmotic system [83]. Qian et al. presented the osmotic energy harvesting in oriented MXene membranes (Figure 9a) [84]. Ions migrating within the interflake channels of horizontally oriented MXene flakes exhibit significantly superior transport behavior compared to those in the perpendicular direction, as witnessed in improvement in ionic conductivity (20.67 S m^−1^ in 1 m NaCl) and diffusion current density (308 A m^−2^ under a tenfold salinity gradient), both of which are orders of magnitude higher than those achieved in the perpendicular direction (Figure 9b,c).

In piezoelectric energy harvesting, the orientation of MXene flakes governs the charge transport pathways and interfacial polarization within piezoelectric polymers, thereby modulating the electromechanical coupling efficiency and overall output performance [85]. Ao et al. reported a temperature–pressure dual‐field regulation strategy to construct an oriented tertiary structure in MXene/poly(vinylidene fluoride) (PVDF) nanocomposites for flexible piezoelectric devices (Figure 9d) [86]. Conventional PVDF‐based piezoelectric polymers suffer from low dipole polarization and random chain orientation, which restricts their piezoelectric response. Here, by applying simultaneous thermal and pressure fields, they achieved a hierarchical orientation involving molecular chains, crystalline domains, and MXene flakes. The horizontally oriented MXene flakes enhance interfacial polarization and charge transport, synergistically promoting dipole alignment in PVDF chains and thereby boosting the overall piezoelectric performance. As a result, the piezoelectric device fabricated from the optimized composite generated an electrical output current nearly 23 times higher than that of the unoriented counterpart (Figure 9e).

Electromagnetic energy harvesting based on MXene conductive coils requires highly horizontal orientation of MXene flakes to ensure superior in‐plane conductivity and efficient electromagnetic coupling [87]. Zhuang et al. reported a spatially confined evaporation strategy to fabricate robust and highly horizontally oriented Ti_3_C_2_T*_x_* MXene films (Figure 9f) [88]. They controlled the wet film thickness (< 60 µm) during solvent evaporation, thereby suppressing the skin effect and preventing defect formation. The resulting films exhibited an extremely dense, smooth, and well‐oriented laminated structure, with a Herman's orientation factor of 0.98–0.99. Consequently, the orientation‐engineered MXene films achieved good tensile strength of ∼707 MPa and electrical conductivity of ∼16 600 S cm^−1^ at a thickness of ∼3.4 µm. As a demonstration, the authors fabricated a flexible MXene near‐field communication antenna, confirming its potential in enable near‐field energy harvesting and signal transmission (Figure 9g,h).

### 4.3 Microelectronics

In microelectronic devices, MXene can be utilized to construct conductive thin films serving as current collectors or electrodes [89]. Unlike conventional metal films deposited by vacuum processes, MXene dispersions can be readily formulated into inks for printing, spin‐coating, or blade coating, offering low‐cost, scalable, and substrate‐compatible fabrication routes that are especially attractive for wafer‐scale microelectronics. Based on this advantage, a new subfield of MXene‐based electronics referred to as “MXetronics” has emerged, highlighting the unique processability and functionality of MXene in microelectronic device integration.

**FIGURE 10:**
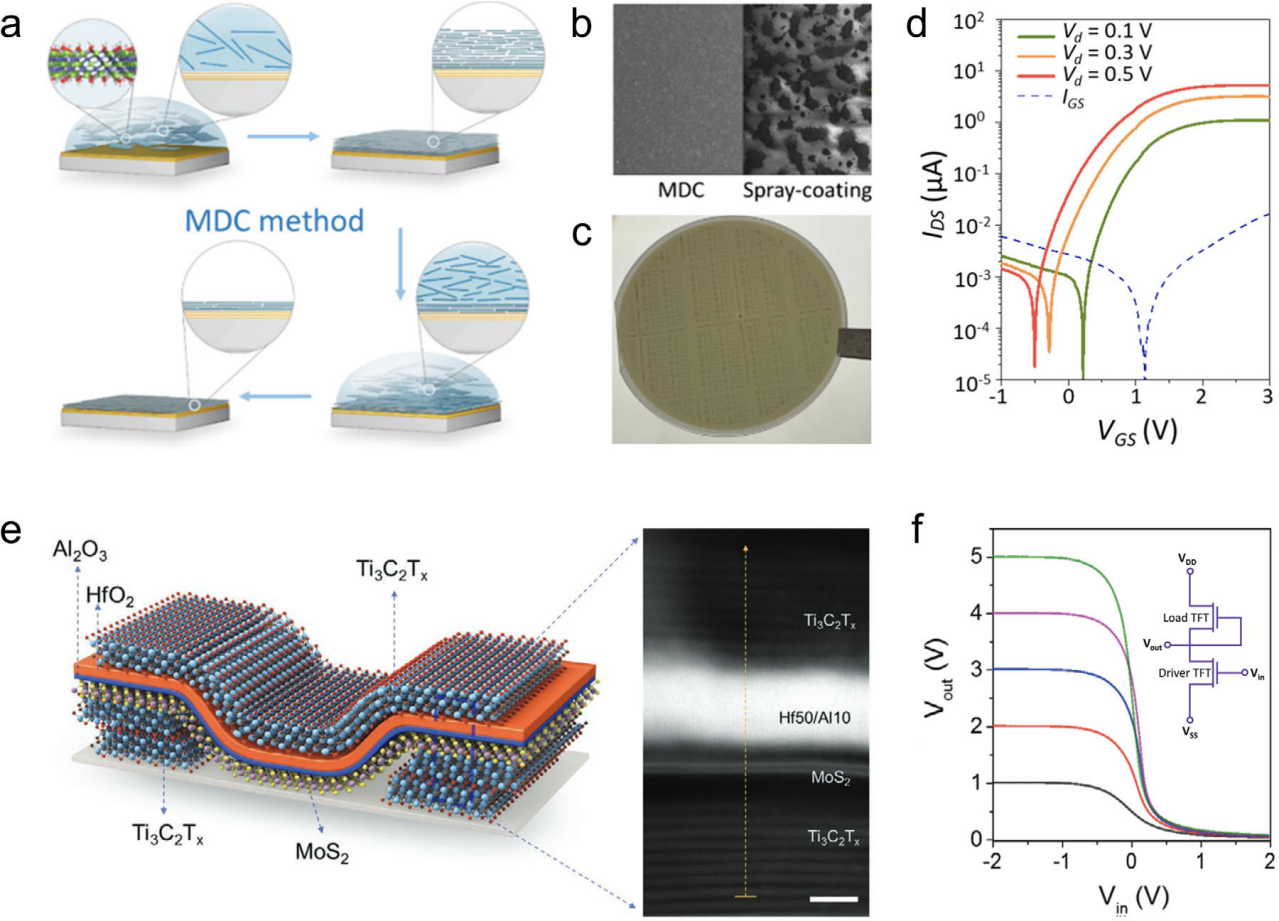
Orientation engineering of MXene flakes for microelectronics. (a) Schematic illustration showing the MDC method in preparing highly horizontally oriented MXene thin film. (b) Morphology of MXene film samples prepared via different techniques. (c) Photo of the wafer‐scale transistor arrays based on such MXene film. (d) Transfer curves of transistors based on such MXene film at different source/drain voltages. Reproduced with permission [44]. Copyright 2023, American Chemical Society. (e) Schematic illustration and cross‐sectional TEM image of the Ti_3_C_2_T*_x_*/MoS_2_ field‐effect transistor. (f) Output voltage transfer curve of the NMOS inverter based on such transistor. Reproduced with permission [90]. Copyright 2021, Wiley‐VCH GmbH.

For microelectronics, highly horizontal orientation of MXene flakes is important. Engineering highly horizontal orientation of MXene flakes within the film can not only minimize interflake junction resistance, reduce surface roughness, and ensure uniform current distribution across the electrode, but also provide enhanced oxidation resistance and environmental stability as the basal planes are predominantly exposed, while the vulnerable edge sites are effectively shielded [89]. Moreover, achieving low‐thickness, high‐orientation MXene films is essential for process compatibility in cleanroom microfabrication workflows, ensuring high pattern resolution, stable adhesion, and reproducible device performance. Guo et al. reported a facile modified drop‐casting (MDC) strategy that enables the direct deposition of highly horizontally oriented, ultrathin Ti_3_C_2_T*_x_* MXene films (∼10 nm) onto hydrophobic MoS_2_ without any harmful surface pretreatment such as UV‐ozone or O_2_‐plasma activation (Figure 10a) [44]. During this process, the negatively charged Ti_3_C_2_T*_x_* flakes electrostatically polarize the MoS_2_ surface, creating interfacial dipoles that promote intimate adhesion and the spontaneous formation of a continuous film during drying. After rinsing, the upper coarse layer is removed while the lower dense MXene layer remains firmly attached, yielding a flat van der Waals interface with an interlayer gap of only ∼6.7 Å. Systematic comparisons show that this MDC process produces much smoother and more conductive films than spray coating (Figure 10b), and that film continuity strongly correlates with substrate dielectric constant (high‐*κ* substrates such as MoS_2_ and HfO_2_ favor stronger electrostatic coupling). Using standard photolithography and lift‐off, the authors patterned wafer‐scale Ti_3_C_2_T*_x_* electrodes on MoS_2_ to fabricate n‐type MXene/MoS_2_ transistors (Figure 10c), which exhibited an average electron mobility of ∼40 cm^2^ V^−1^ s^−1^, on/off ratio of ∼1×10^5^, and subthreshold swings below 200 mV dec^−1^ with excellent device uniformity across the wafer (Figure 10d). Xu et al. reported a two‐step spray‐annealing strategy for producing high‐quality horizontally oriented Ti_3_C_2_T*_x_* MXene films and their integration with wafer‐scale MoS_2_ to realize high‐yield, solution‐processed 2D integrated circuits (Figure 10e) [90]. The 400°C vacuum annealing treatment removes the intercalated water and enhances the horizontal orientation of the film, as evidenced by the sharpened and shifted (0002) family peaks of MXene in XRD spectra. This simple yet effective two‐step process dramatically increased the film conductivity from ∼6300 S cm^−1^ to ∼11 000 S cm^−1^. The optimized MXene/MoS_2_ transistor arrays achieve ∼96% device yield with on/off ratios exceeding 10^6^ and minimal leakage currents. Furthermore, functional circuits including rectifiers, negative‐channel metal oxide–semiconductor (NMOS) inverters, and voltage‐shift NMOS inverters are demonstrated based on such engineered MXene films (Figure 10f).

### 4.4 Optoelectronics

Transparent conductive electrodes (TCEs), serving as fundamental current collectors and light‐transmitting layers in optoelectronic systems, represent another key application scenario where the orientation of MXene flakes is essential in determining their transparency and conductivity [91]. For TCE applications, horizontal MXene orientation is important, as it can optimize in‐plane electrical percolation at minimal thickness, thereby supporting high transmittance together with low sheet resistance. In TCEs, film thickness is a key parameter, as increasing the thickness improves electrical conductivity but compromises optical transmittance, and vice versa. While TCE design often entails a balance between transparency and conductivity, horizontally oriented MXene flakes can help mitigate this trade‐off via enabling thinner films to retain sufficient conductivity. Such orientation‐optimized MXene films provide a promising foundation for developing devices such as smart windows, flexible displays, and wearable optoelectronic platforms.

**FIGURE 11:**
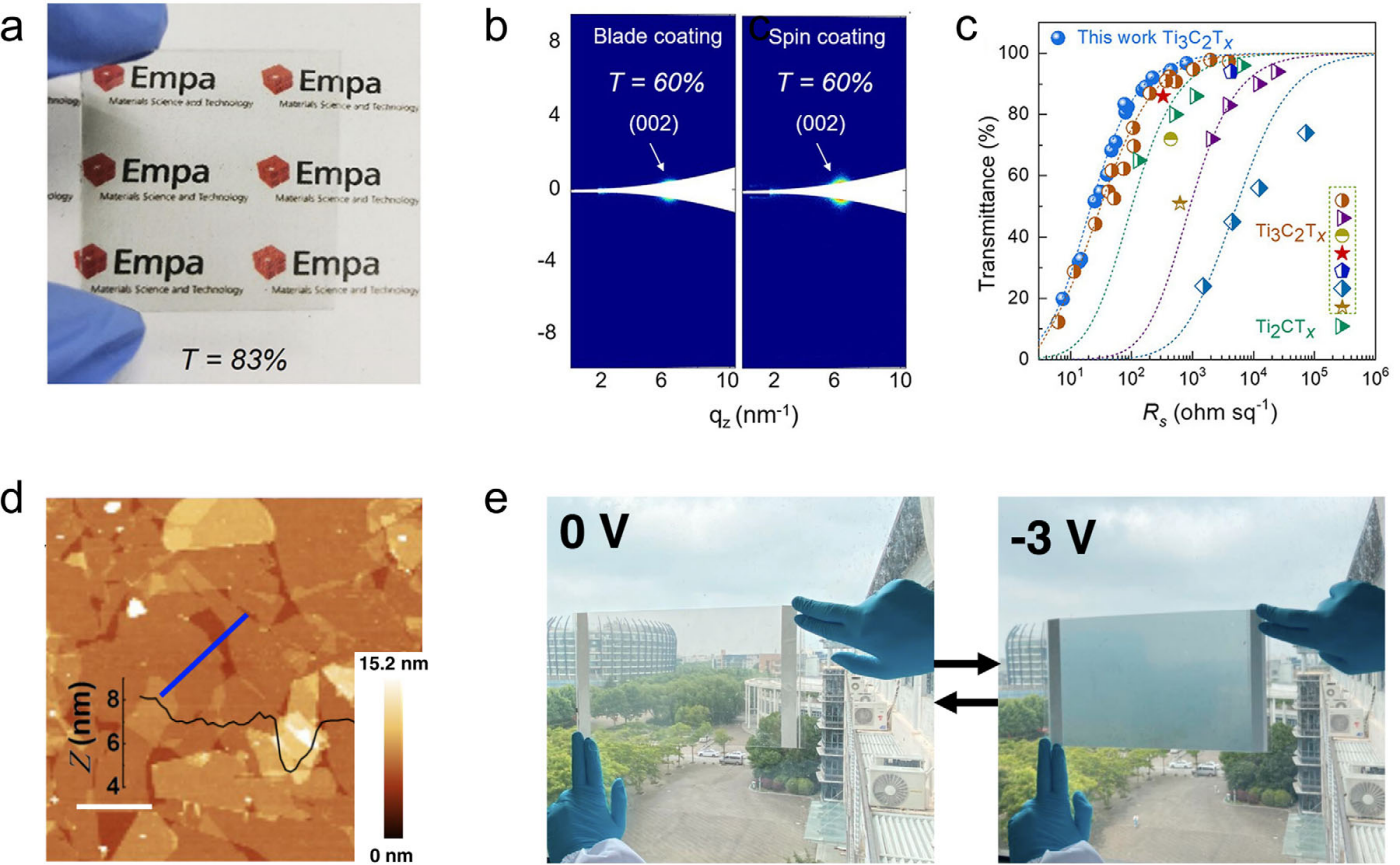
Orientation engineering of MXene flakes for optoelectronics. (a) Photo of a blade‐coated ultralarge‐flake Ti_3_C_2_T*_x_* film fabricated from ultralarge flakes on a glass substrate. (b) GISAXS pattern of MXene films prepared via different techniques. (c) Relationship between optical transmittance and sheet resistance for MXene TCE films with varying thicknesses. Reproduced under the terms of the CC‐BY 4.0 license [92]. Copyright 2023, The Authors. Published by American Chemical Society. (d) AFM image and corresponding height profile of the assembled Ti_3_C_2_T*_x_* thin film. (e) The operation of MXene/TiO_2_‐based electrochromic device employing MXene TCEs on a glass window. Reproduced under the terms of the CC‐BY 4.0 license [93]. Copyright 2021, The Authors. Published by Springer Nature.

Guo et al. presented the rational design of Ti_3_C_2_T*_x_* MXene TCEs by engineering ultralarge single‐layer Ti_3_C_2_T*_x_* flakes with an average lateral size of ∼12.2 µm and applying shear‐induced orientation through blade coating to obtain compact, highly oriented MXene films (Figure 11a) [92]. The use of ultralarge MXene flakes effectively minimizes interflake resistance and mitigates percolation effects at high transmittance, thereby enabling good TCE performance. GISAXS characterizations further confirm dense laminar stacking and good flake orientation of these ultralarge MXene flakes, yielding a Herman's orientation factor of 0.78 for the blade‐coated film (Figure 11b). Good TCE performance is achieved on such MXene thin film, exhibiting a high transparency of 96.7% along with a sheet resistance of 800 Ω sq^−1^ (Figure 11c). Leveraging these properties, the authors fabricated transparent Joule heaters that reached ∼93°C at 5 V with uniform thermal distribution. Li et al. developed electrochromic devices based on self‐assembled horizontally oriented 2D TiO_2_/Ti_3_C_2_T*_x_* heterostructures [93]. The oriented MXene structure is obtained via a liquid–liquid interfacial assembly technique, enabling large‐area, nanometer‐thick films with highly oriented and interconnected 2D flakes (Figure 11d). A mild oxidation treatment is employed to convert the Ti_3_C_2_T*_x_* MXene into TiO_2_ and assemble it into oriented film via the same liquid‐phase process. Based on the oriented TiO_2_/Ti_3_C_2_T*_x_* heterostructure, flexible electrochromic devices are fabricated, exhibiting fast response, high coloration efficiency, and excellent scalability for large‐area smart window applications (Figure 11e).

### 4.5 Flexible Electronics

Flexible electronic devices demand materials that not only exhibit excellent electrical performance but also maintain mechanical integrity under repeated bending, stretching, or twisting [94, 95]. MXene, with its intrinsic flexibility and high conductivity, is promising candidates for such applications [96, 97]. Importantly, the orientation of MXene flakes within flexible structures significantly affects their electrical robustness and strain tolerance. For flexible electrodes, horizontal orientation of MXene flakes is important, as it can help build a strain‐tolerant conductive network. Horizontally oriented MXene membranes offer more continuous conductive pathways and better load distribution during deformation, thereby enhancing both conductivity retention and mechanical durability. While flexible electronics cover a wide spectrum of applications such as energy storage, sensing, and actuation, this section specifically highlights orientation‐engineered MXene structures as flexible electrodes, which serve as the fundamental components enabling high‐performance flexible electronic devices.

**FIGURE 12:**
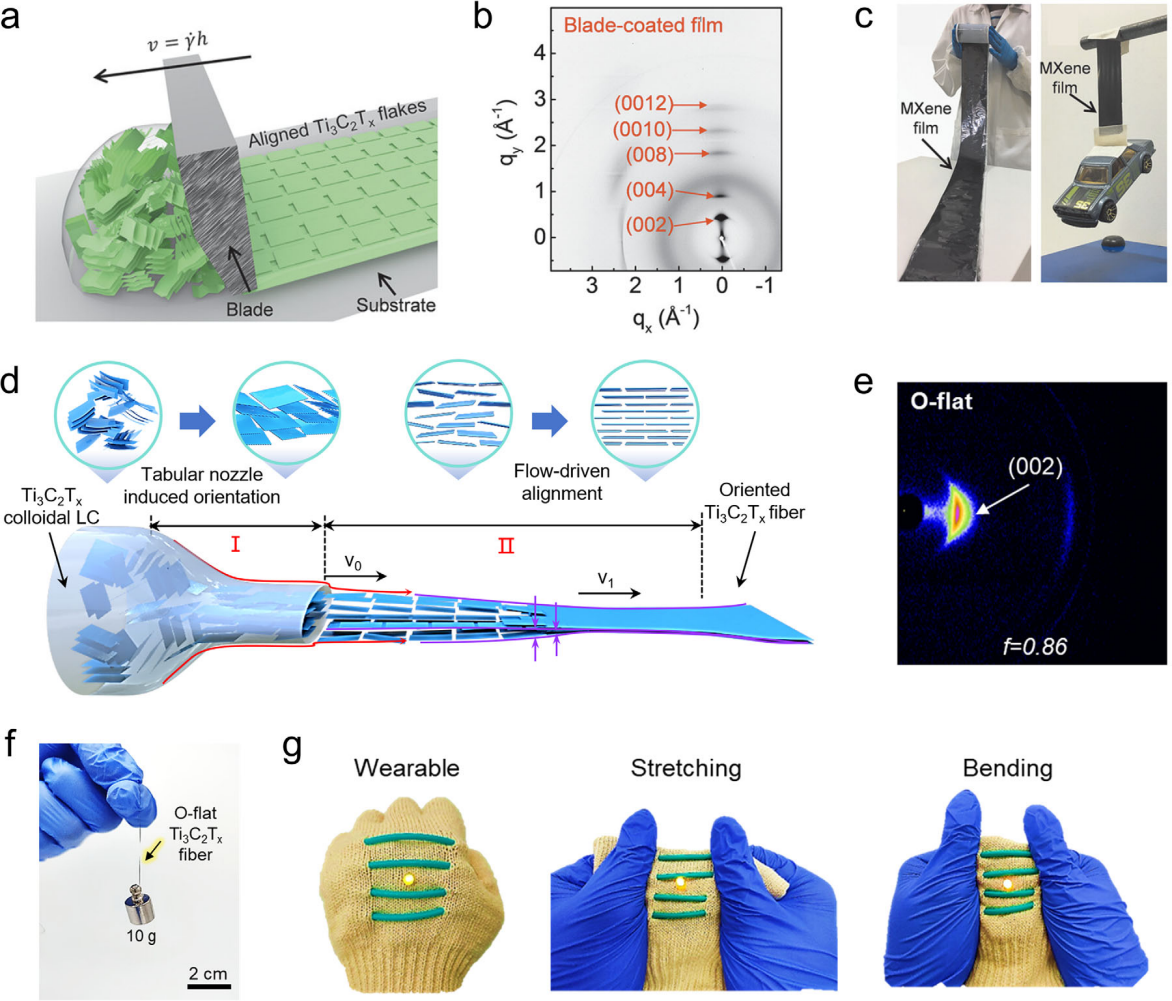
Orientation engineering of MXene flakes for flexible electronics. (a) Schematic illustration showing the horizontally oriented MXene film engineered via a blade coating process. (b) SAXS‐WAXS pattern showing the highly oriented MXene film. (c) Photos showing such MXene film at large scale and for lifting a toy car. Reproduced with permission [64]. Copyright 2020, Wiley‐VCH GmbH. (d) Schematic illustration showing the orientation evolution of a MXene fiber during a wet‐spinning process. (e) WAXS pattern of the O‐flat MXene fiber. Photos showing the O‐flat MXene fiber in (f) hanging a 10 g weight and (g) assembling a flexible supercapacitor for powering an LED in a glove. Reproduced with permission [67]. Copyright 2021, American Chemical Society.

Representative examples of films and fibers based on orientation‐engineered MXene structures, which exhibit great potential as flexible electronic electrodes, are summarized below. Zhang et al. reported a scalable blade‐coating strategy to fabricate free‐standing, strong, and highly horizontally oriented Ti_3_C_2_T*_x_* MXene films without any additives (Figure 12a) [64]. By preselecting large Ti_3_AlC_2_ MAX particles (>10 µm), they obtained single‐layer MXene flakes with an average lateral size of ∼10 µm that form lyotropic liquid‐crystal dispersions exhibiting shear‐thinning behavior. During blade coating, the shear force orients the large MXene flakes parallel to the film plane, yielding densely stacked structures with a highly orientation factor of 0.75 (Figure 12b). As a result, the blade‐coated films achieved a high tensile strength of ∼570 MPa at 940 nm thickness, and a high electrical conductivity of ∼15 100 S cm^−1^ at 214 nm thickness. The films also demonstrated excellent flexibility, showing a minimal resistance change (< 1%) after 5000 bending cycles, and exhibited great potential for large‐scale preparation and mechanical load bearing under practical flexible conditions, highlighting their strong promise as flexible conductive electrodes (Figure 12c).

Li et al. presented a highly horizontally oriented MXene fiber using a wet‐spinning process with a flat spinneret and postcoagulation drawing treatment (Figure 12d) [67]. With such orientation engineering, the as‐developed highly oriented Ti_3_C_2_T*_x_* fibers (O‐flat fiber) exhibit a Herman's orientation factor of 0.86 (Figure 12e), which leads to good mechanical strength of 118 MPa and electrical conductivity of 7200 S cm^−1^, much better than the nonoriented MXene fibers. Benefiting from its outstanding mechanical and electrical properties, the O‐flat MXene fiber exhibits significant promise as a fiber‐shaped conductive electrode for flexible electronic devices. It is capable of lifting loads many times its own weight and can function as the electrode of flexible fiber supercapacitors, which can be seamlessly integrated into wearable textiles such as gloves to power LEDs (Figure 12f,g).

### 4.6 Sensors

MXene has also found widespread applications in sensor technologies, owing to their excellent electrical conductivity, large surface area, and abundant functional terminations that enable highly efficient signal transduction [98]. The orientation engineering of MXene is instrumental in tailoring its sensing mechanisms, which can be broadly categorized into resistive‐type (electron‐conduction‐based) and ionic‐type (ion‐transport‐based) sensors. For sensing applications, the preferred MXene orientation is mechanism‐dependent: horizontal orientation mainly enhances in‐plane electronic percolation in resistive‐type sensors, whereas vertical architectures are more effective for ionic‐type sensing where ion transport and interfacial access dominate the sensing process.

**FIGURE 13:**
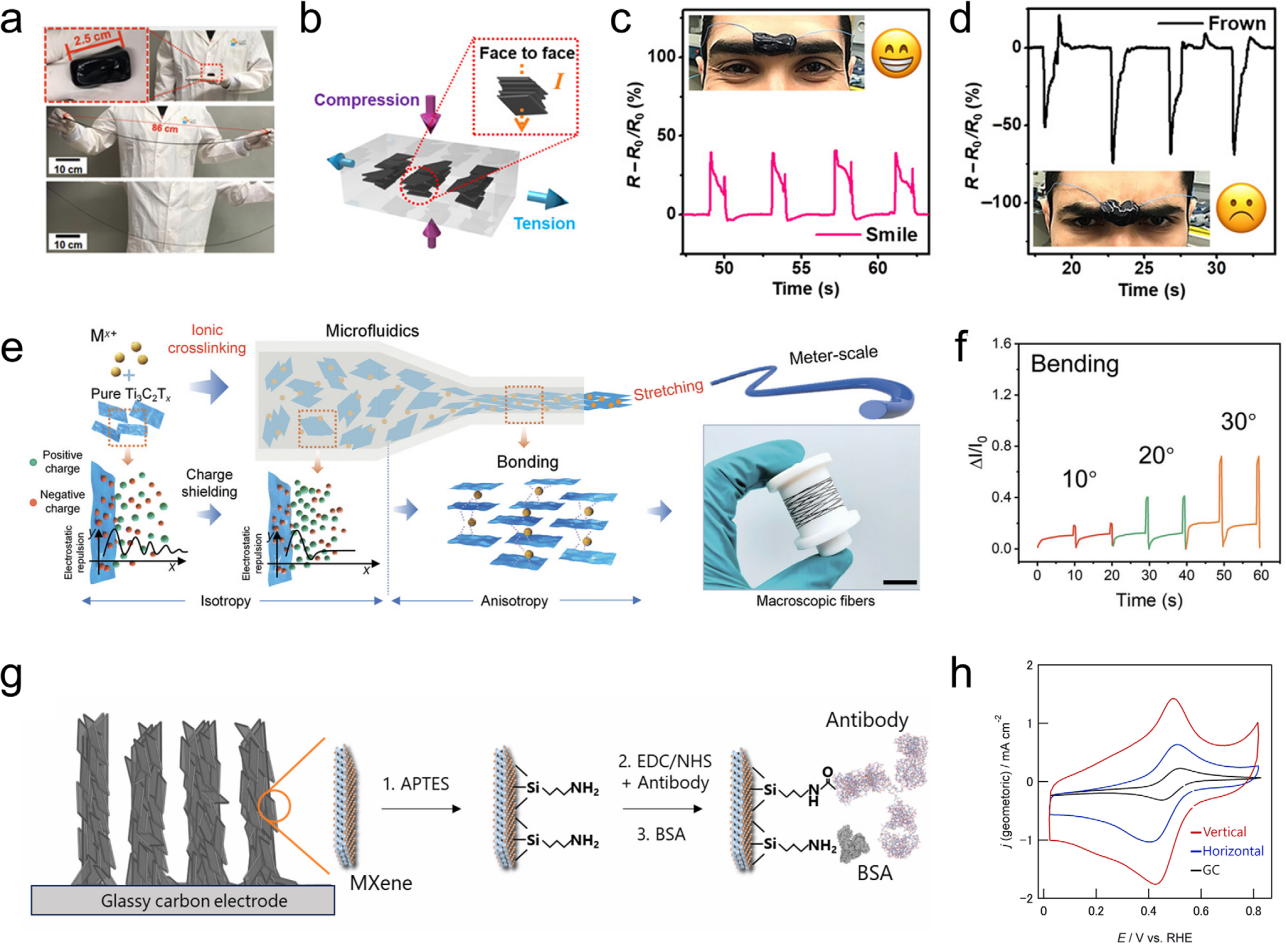
Orientation engineering of MXene flakes for sensors. (a) Photos showing the MXene/PVA hydrogel and its stretchability. (b) Schematic illustration showing the electromechanical response mechanism of the MXene/PVA hydrogel. Resistance variation of the MXene/PVA hydrogel in response to (c) smile and (d) frown facial expressions. Reproduced with permission [72]. Copyright 2018, The American Association for the Advancement of Science. (e) Schematic illustration showing the fabrication process of highly oriented MXene fibers reinforced by ionic interactions in the spinning dope. (f) Sensing performance of the MXene fiber during finger bending at various angles. Reproduced with permission [45]. Copyright 2024, Wiley‐VCH GmbH. (g) Schematic illustration of a biosensing platform based on an antibody‐functionalized, vertically oriented MXene electrode. APTES, EDC, NHS, and BSA refer to 3‐aminopropyltriethoxysilane, *N*‐(3‐Dimethylaminopropyl)‐*N*′‐ethylcarbodiimide hydrochloride, *N*‐Hydroxysuccinimide, and bovine serum albumin, respectively. (h) CV curves of different electrodes tested in phosphate‐buffered saline (PBS) containing 5 mm [Ru (NH_3_)_6_]^3+^. GC refers to glassy carbon electrode. Reproduced with permission [101]. Copyright 2024, The Authors. Published by Elsevier B.V.

In resistive sensors, horizontally oriented MXene flakes can form continuous conductive pathways within the plane, which ensures efficient electron percolation and minimizes interflake tunneling barriers [99]. When external mechanical deformation (e.g., strain, pressure, or bending) occurs, the interflake spacing and overlap between flakes dynamically change, leading to reversible modulation of resistance. This orientation‐dependent conductive network enables MXene‐based flexible strain or pressure sensors to achieve high sensitivity, wide detection range, and outstanding durability. Zhang et al. developed a highly stretchable, self‐healable, and multifunctional hydrogel‐based strain sensor by integrating MXene flakes into a PVA hydrogel matrix (Figure 13a) [72]. They revealed that the dynamic reorientation of initially disoriented MXene flakes under mechanical loading is central to the electromechanical response of the hydrogel (Figure 13b). Under tensile strain, the interflake distance increases and the number of conductive junctions decreases, resulting in higher resistance. In contrast, compressive strain forces the flakes closer together, transforming many surface‐edge contacts into face‐to‐face junctions that markedly enhance electrical conductivity and lower resistance. This direction‐dependent reorientation of MXene flakes enables precise recognition of facial expressions based on distinct electrical signal patterns. When the skin undergoes tensile deformation (such as smiling), the enlarged interflake spacing disrupts conductive pathways, causing an increase in resistance (Figure 13c). Conversely, during compressive deformation (such as frowning), the flakes approach one another, forming more conductive contacts and thereby decreasing resistance (Figure 13d). More recently, Li et al. reported a universal multivalent ion crosslinking strategy to fabricate highly oriented and mechanically robust MXene nanofluidic fibers with simultaneously enhanced electronic and ionic conductivities (Figure 13e). By regulating the electrostatic interactions between negatively charged Ti_3_C_2_T*_x_* flakes through the introduction of multivalent cations, the authors achieved tunable rheology and induced the gelation of MXene liquid crystals suitable for wet‐spinning. During fiber formation, strong shear and extensional flow fields orient the MXene flakes along the fiber axis, resulting in dense packing and high orientation (Herman's factor up to 0.84). Consequently, the Zn^2+^–Ti_3_C_2_T*_x_* fibers exhibit exceptional performance with an electrical conductivity of 11 200 S cm^−1^ and tensile strength of 252 MPa. When integrated as flexible resistive sensors, the oriented Zn^2+^–Ti_3_C_2_T*_x_* fibers precisely detect finger‐bending motions with rapid, reversible current changes (Figure 13f).

On the other hand, ionic‐type sensors rely on the interflake transport of ions or molecules in the MXene structure, and thus the vertical MXene orientation is essential [100]. The vertically oriented flakes create open, through‐plane channels that allow ions to penetrate and diffuse efficiently across the interflake channels, significantly accelerating the electrochemical response. Changes in humidity, gas concentration, or analyte content modulate the ionic flux or redox behavior within these vertically oriented channels, giving rise to measurable current or potential shifts. Hideshima et al. reported Ti_3_C_2_T*_x_* MXene bioelectrodes fabricated via freeze‐drying‐assisted electrophoretic deposition, enabling highly porous and vertically oriented architectures for ultrasensitive electrochemical protein detection (Figure 13g) [101]. Compared to conventional horizontally oriented MXene films suffer from low porosity and limited ion diffusion, the vertical oriented film exhibits open ion channels, large surface area, and efficient charge transfer, as evidenced by a ∼2.5 times higher capacitance and smaller redox peak polarization in the cyclic voltammetry (CV) tests (Figure 13h). After antibody functionalization, the vertically oriented MXene electrode could specifically detect food‐allergenic proteins, such as the buckwheat allergen BWp16, showing a clear and concentration‐dependent electrochemical response.

### 4.7 Soft Actuators

With the rapid evolution of soft robotics and intelligent materials, stimuli‐responsive actuators have emerged as indispensable components in wearable electronics, biomedical devices, and adaptive systems. Among various functional materials, MXene has garnered significant attention owing to its exceptional electrical conductivity, mechanical flexibility, high surface area, and outstanding photothermal/electrothermal conversion efficiency [102]. These properties make MXene a promising candidate for next‐generation soft actuators with high responsivity, tunable deformation, and multifunctional integration. Beyond intrinsic material properties, the orientation of MXene flakes within composite matrices has recently been recognized as a critical structural parameter dictating the actuation performance. Controlled flake orientation can tailor the anisotropic transport of ions, electrons, and heat, enhance mechanical coupling across interfaces, and introduce programmable deformation modes under external stimuli. For soft actuators, vertical orientation is of great importance, as their abundance interlayer space can enable fast response and large deformation. Moreover, combining vertical and horizontal MXene orientations into a Janus architecture is also meaningful for soft actuators, as it can realize programmable anisotropic deformation of the actuator.

**FIGURE 14:**
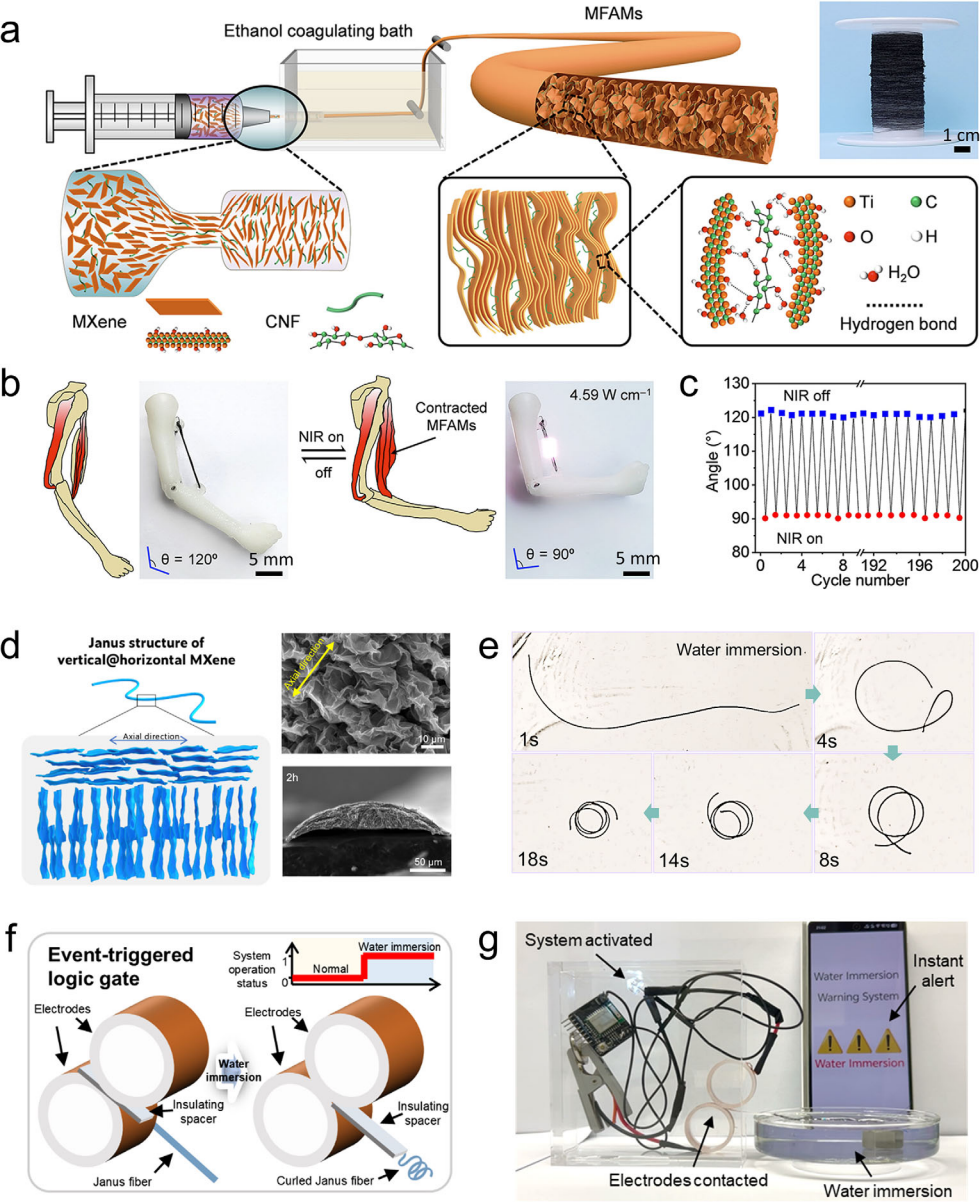
Orientation engineering of MXene flakes for soft actuators. (a) Schematic illustration showing the preparation process of MXene/CNF fiber with vertical orientation of MXene flakes. (b) Schematic illustration and photos showing the application of such vertical fiber as artificial muscle under NIR stimulation and (c) the corresponding cycling data. Reproduced with permission [103]. Copyright 2025, The American Association for the Advancement of Science. (d) Schematic illustration and SEM images showing the MXene fiber with Janus orientation. (e) Photos showing the curling process of the Janus MXene fiber under water immersion. (f) Schematic illustration showing the application of such Janus fiber for actuating an event‐triggered logic gate and (g) the alarming of the system in practical water immersion situation. Reproduced with permission [104]. Copyright 2025, Wiley‐VCH GmbH.

Vertically oriented MXene structures can facilitate rapid ion and molecule transport through open interflake channels, and the consequent abundant spaces are capable of accommodating macroscopic structural expansion and contraction. Fu et al. developed MXene/cellulose nanofiber (CNF) composite fibers via a wet‐spinning process, where the MXene flakes were vertically oriented by precisely engineering the flow field during spinning (Figure 14a) [103]. The resulting fibers exhibit thermal actuation driven by a temperature‐induced reorganization of nanoconfined hydrogen bond networks formed between MXene flakes, CNFs, and intercalated water molecules. Based on these fibers, the authors constructed MXene fiber artificial muscles (MFAMs), which demonstrate highly reversible near‐infrared (NIR)‐responsive tensile contraction behavior (Figure 14b,c).

Engineering the orientation of MXene flakes into a Janus configuration, where half of the fiber cross‐section exhibits horizontal orientation and the other half is vertically oriented, is a more advanced concept in soft actuating. Upon external stimulation, the vertically oriented region undergoes greater axial expansion than the horizontal region, resulting in giant asymmetric deformation across the fiber cross‐section. This anisotropic response can even induce helical twisting or bending deformation, offering new opportunities for programmable, multimodal actuation. Very recently, Li et al. presented this concept with a Janus‐oriented MXene fiber via combining an engineered spinning process with subsequent asymmetric drying (Figure 14d) [104]. The horizontally oriented domains ensure high mechanical strength of the MXene fiber, whereas the vertically oriented side enables strong responsiveness to external water stimuli. Furthermore, the resulting structural anisotropy induces asymmetric swelling under water stimuli, leading to pronounced curling and helical deformation with a record length change of up to 2100% (Figure 14e). Employing this giant and reversible actuation, they further demonstrated a proof‐of‐concept water‐immersion warning system, in which the Janus MXene fiber functions as an event‐triggered logic gate to control both light emission and Bluetooth signaling, while effectively preventing false triggering under high humidity (Figure 14f,g).

### 4.8 Electromagnetic Interference Shielding

EMI shielding refers to a technology that uses materials or structural designs to block, reflect, or absorb electromagnetic waves, thereby preventing electronic devices from generating or experiencing EMI. MXene has emerged as a promising candidate for EMI shielding, primarily due to its intrinsic metallic conductivity, which enables efficient reflection of incident electromagnetic waves [105, 106, 107]. Horizontal orientation of MXene flakes generally plays a key role in determining their EMI shielding performance. Horizontally oriented MXene flakes can construct highly continuous in‐plane conductive pathways, significantly enhancing the reflection of incident electromagnetic waves [108]. In addition, the laminated structures of the horizontally oriented MXene flakes can introduce multiple internal reflections and extend the propagation path, thereby further enhancing overall EMI shielding performance. Moreover, compared with randomly oriented assemblies that may contain interflake voids and cracks, horizontally oriented architectures form more uniform and compact lamellar interfaces, thereby suppressing electromagnetic wave penetration and leakage.

**FIGURE 15:**
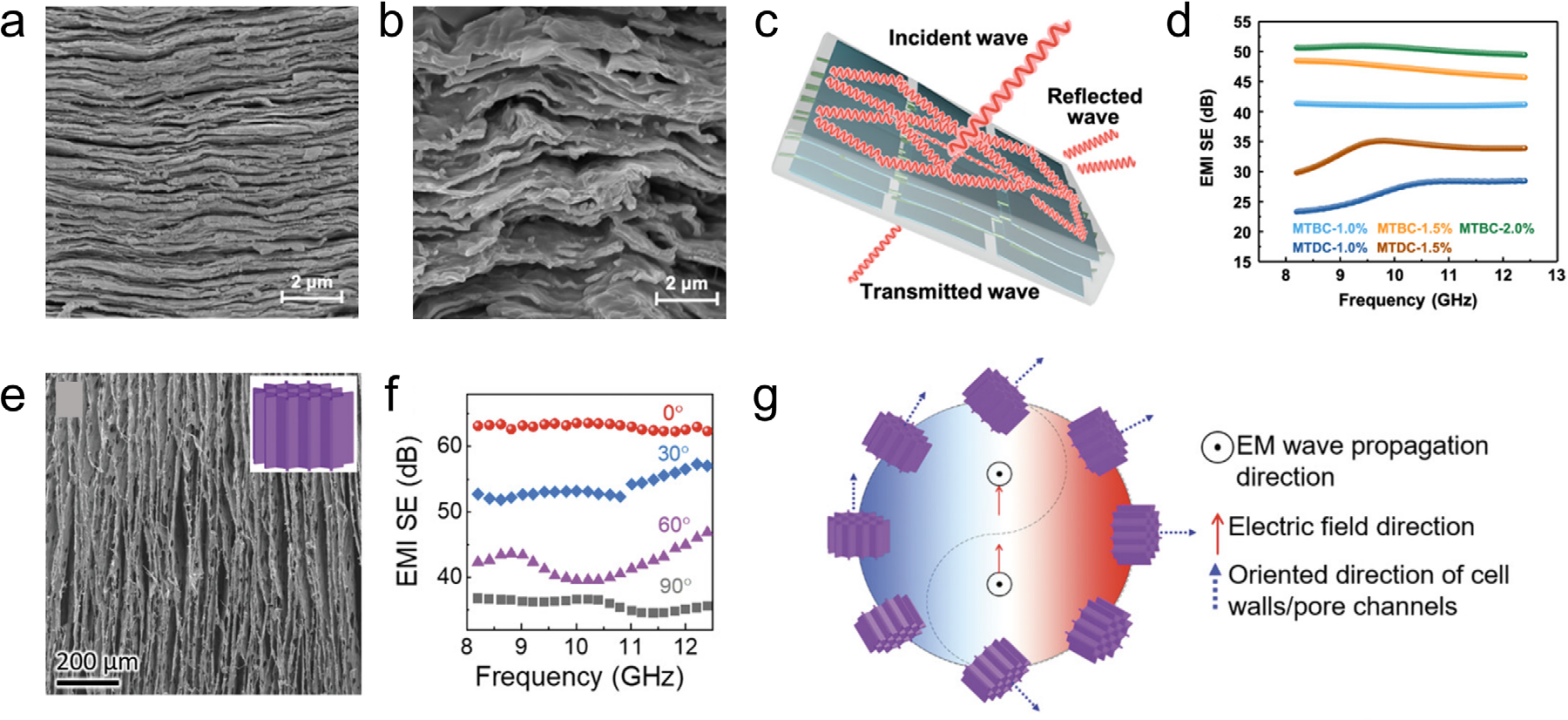
Orientation engineering of MXene flakes for electromagnetic interference shielding. SEM images showing the MXene/cellulose nanofiber composite film prepared via (a) blade coating and (b) drop casting. (c) Schematic illustration showing the EMI shielding mechanism of highly horizontally oriented MXene/cellulose nanofiber composite film. (d) EMI shielding performance of different MXene samples. Reproduced with permission [109]. Copyright 2022, American Chemical Society. (e) SEM image of vertically oriented MXene/cellulose nanofibrils aerogel. (f) EMI shielding performance and (g) schematic illustration showing the angle‐dependent EMI shielding of such structure. Reproduced under the terms of the CC BY 4.0 license [110]. Copyright 2020, The Authors. Published by Wiley‐VCH GmbH & Co. KGaA, Weinheim.

Feng et al. developed a rheology‐guided assembly strategy to fabricate horizontally oriented MXene/cellulose nanofiber composite films for EMI shielding [109]. By optimizing the rheological behavior of MXene/cellulose nanofiber composites for shear‐induced blade coating, a large‐area (500 cm^2^) MXene film was prepared, exhibiting enhanced horizontal orientation compared with the randomly oriented drop‐casted counterpart (Figure 15a,b). The highly oriented blade‐coated MXene film exhibits an electrical conductivity of 46 685 S m^−1^, nearly seven times higher than that of the randomly stacked drop‐casted film (6929 S m^−1^), which consequently results in a much stronger EMI shielding performance derived from the enhanced horizontal orientation (Figure 15c,d). Furthermore, Zeng et al. reported the design of ultralight MXene/cellulose nanofibril biomimetic aerogels for orientation‐tunable EMI shielding performance [110]. The orientation of Ti_3_C_2_T*_x_* MXene/cellulose is engineered into a vertical mode via a unidirectionally freeze‐cast process (Figure 15e). The resulting aerogels exhibit low densities yet outstanding EMI shielding effectiveness (SE), reaching 74.6 dB at a density of 8.0 mg cm^−3^. Importantly, the study reveals that the shielding performance can be precisely tuned by changing the orientation angle between the cell walls and the incident electric field, where maximum SE occurs in the parallel direction and minimal at the perpendicular direction (Figure 15f,g). This orientation‐dependent EMI shielding mechanism was later verified in MXene/wood composites as well [111]. These studies demonstrate that simply altering the orientation of MXene flakes allows the shielding performance to be enhanced or weakened through orientation engineering, revealing the potential of MXene orientation engineering for applications that need switchable EMI shielding behaviors.

### 4.9 Water Treatment Applications

MXene has emerged as promising materials for water purification and desalination due to their high surface area, tunable interflake spacing, and abundant surface terminations that interact with various contaminants [112, 113]. In this context, orientation engineering is of great importance in regulating mass transport pathways and enhancing selectivity in membrane‐based separation systems. For water‐treatment membranes, the optimal MXene orientation should be selected based on the performance metrics prioritized for the target application. Horizontal orientation is typically favored for high selectivity via well‐defined lamellar nanochannels, whereas vertical orientation is often pursued to improve through‐thickness permeability and mitigate transport resistance.

**FIGURE 16:**
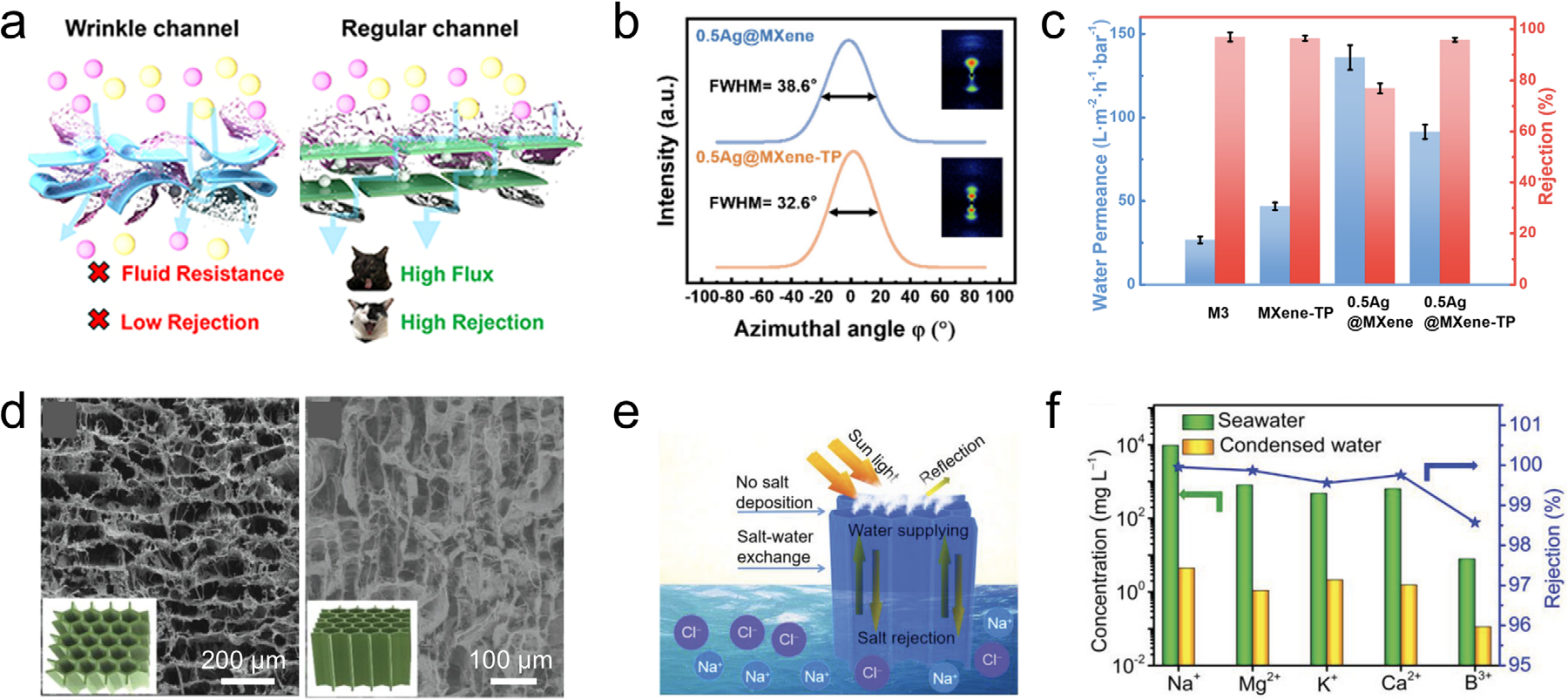
Orientation engineering of MXene flakes for water treatment applications. (a) Schematic illustration of oriented and nonoriented MXene membranes for nanofiltration. (b) Azimuthal intensity plots extracted from GIWAXS patterns of different membranes. (c) Nanofiltration performance of different membranes tested with Rhodamine B solution. Reproduced with permission [115]. Copyright 2025, AIP Publishing. (d) SEM images showing the morphology of vertically oriented MXene‐based aerogel. (e) Schematic illustration showing the salt rejection mechanism and overall process of solar energy‐driven seawater desalination. (f) Ion rejection of East China Sea seawater and condensed water after solar‐driven desalination using such aerogel. Reproduced with permission [116]. Copyright 2023, Wiley‐VCH GmbH.

Horizontally oriented MXene flakes form densely packed lamellar structures with narrow and tortuous channels, which are effective for rejecting large organic molecules or selectively screening ions based on size or charge [114]. Luo et al. reported horizontally oriented MXene‐based nanofiltration membranes with enhanced channel regularity and permeability, in contrast to the irregular nanochannels and stacking defects typically found in conventional MXene membranes (Figure 16a) [115]. To achieve this, the authors premodified Ti_3_C_2_T*_x_* flakes with tea polyphenols whose abundant hydroxyl groups served as antioxidants and spacers to prevent Ag^+^‐induced aggregation, suppress oxidation, and flatten the MXene flakes. Consequently, the optimized MXene membranes exhibit highly ordered lamellar structures with reduced corrugations and more uniform nanochannels, as verified by GIWAXS study (Figure 16b). Under such orientation advantages, the membrane exhibited significantly improved water permeability far exceeding that of pristine MXene membranes, while maintaining high dye rejection efficiencies (95.85% for Rhodamine B and 97.30% for Congo Red), as shown in Figure 16c.

Vertically oriented architectures create more open and straight‐through pathways, enabling faster water flux and reduced transport resistance, which is particularly beneficial for solar‐driven water desalination and purification. Wang et al. presented vertically oriented MXene‐based aerogel with compositing polydopamine, hydroxyapatite nanowires, and polyacrylamide/polyvinyl alcohol binders [116]. The unidirectional freeze‐drying process endows the MXene‐based aerogel with a hierarchical honeycomb structure featuring vertically oriented microchannels (Figure 16d). Such unique low‐tortuosity vertical channels enable rapid upward water transport while simultaneously allowing salt ions accumulated on the surface to diffuse back downward into the bulk solution, thereby ensuring continuous and efficient salt‐resistant operation (Figure 16e). Real seawater from the East China Sea was desalinated using the aerogel, achieving over 98.5% ion rejection, with the concentrations of Na^+^, Mg^2+^, K^+^, Ca^2+^, and B^3+^ reduced by three orders of magnitude, demonstrating its excellent desalination efficiency (Figure 16f).

### 4.10 Anticorrosion

**FIGURE 17:**
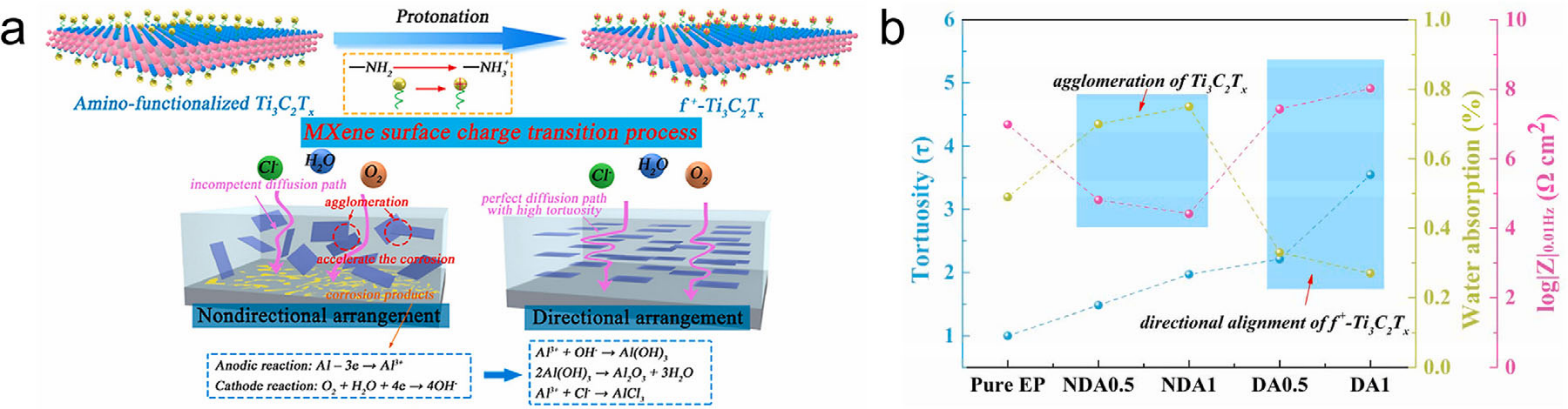
Orientation engineering of MXene flakes for anticorrosion. (a) Schematic illustration showing the anticorrosion mechanism of the oriented MXene coating layer. (b) Corrosion resistance of different samples. EP, NDA, and DA refer to epoxy, nondirectionally arranged, and directionally arranged. The number refers to the weight percentage of MXene in the composite. Reproduced with permission [119]. Copyright 2021, Elsevier Ltd.

In anticorrosion protection, MXene‐based coatings can be applied to substrates as barrier layers, where the horizontal orientation of MXene flakes is a key structural requirement. When MXene flakes are stacked parallel to the substrate, they form a compact, layered barrier that effectively blocks the penetration of corrosive species such as water molecules, oxygen, and ions by creating a long and tortuous diffusion pathway [117]. This horizontal orientation minimizes defects and pinholes, thereby enhancing the impermeability and corrosion resistance of the coating layer [118]. In contrast, vertically oriented or randomly distributed MXene flakes can create open channels that accelerate the diffusion of electrolytes and expose reactive edges, resulting in faster degradation. Fan et al. reported horizontal orientation of Ti_3_C_2_T*_x_* MXene flakes in epoxy coatings via an electrophoretic deposition process, aiming to maximize their anticorrosion and antiwear performance (Figure 17a) [119]. They first modified Ti_3_C_2_T*_x_* flakes with polyethyleneimine and then protonated them to obtain positively charged MXene flakes (f^+^‐Ti_3_C_2_T*_x_*), which can migrate and rotate under an external electric field. During electrophoretic deposition, these charged flakes move toward the cathode and form a highly horizontally oriented barrier layer, which enhances barrier tortuosity and interfacial compatibility, suppressing the penetration of corrosive ions and improving coating integrity. As a result, the optimized coating with 1 wt% f^+^‐Ti_3_C_2_T*_x_* exhibits decreased water absorption and enhanced lowest frequency impedance compared to randomly oriented samples, demonstrating superior long‐term corrosion protection (Figure 17b).

## 5 Summary and Outlook

From individual MXene flakes to integrated MXene‐based devices, MXene must be assembled into well‐defined structures, where the orientation of MXene flakes plays a decisive role in governing their overall performance. This review systematically discusses the orientation‐dependent properties of MXene assemblies, the techniques for characterizing MXene orientation, the fabrication strategies for oriented MXene structures, and the diverse application scenarios with optimized MXene orientation. As an emerging yet crucial direction within the expanding MXene research landscape, orientation engineering offers a powerful handle to bridge structure–property–function relationships in both fundamental studies and device‐level implementations. Although notable progress has been achieved in understanding the basics of MXene orientation engineering, there is still much remaining to be explored. Apart from the optimization of fundamental MXene synthesis chemistries (e.g., more MXene compositions, greener synthesis, mass production), future investigations on MXene orientation engineering are expected to focus on the following key aspects.

**FIGURE 18:**
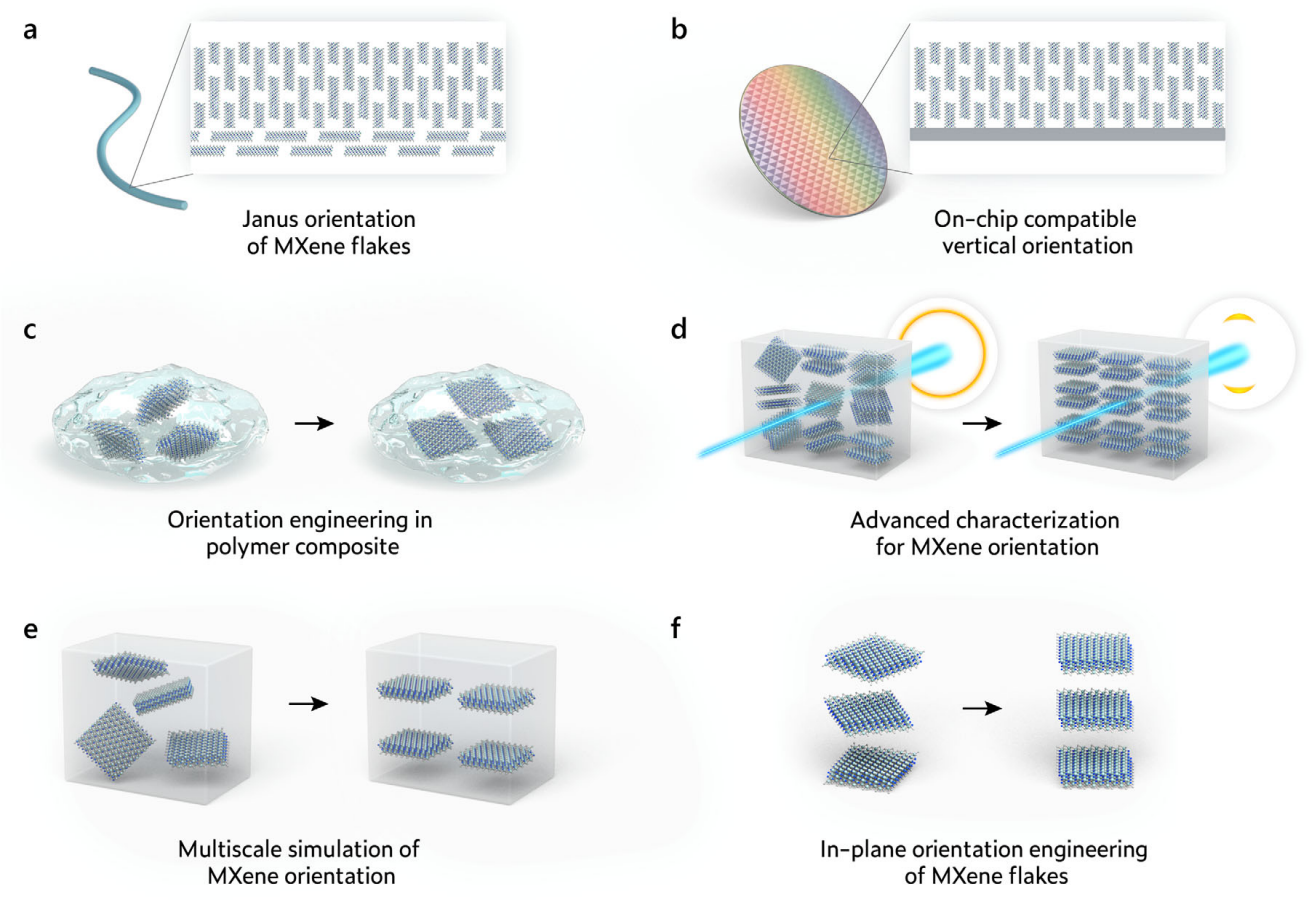
Future perspectives on orientation engineering of MXene flakes regarding the following aspects: (a) Janus orientation, (b) wafer‐scale vertical orientation, (c) orientation engineering in polymer composite, (d) advanced orientation characterization, (e) multiscale orientation simulation, and (f) in‐plane orientation.

Janus orientation of MXene flakes with precise spatial orientation distribution (Figure 18a). As mentioned above, the horizontal orientation of MXene flakes offers high in‐plane conductivity and mechanical strength but suffers from limited ion accessibility, while vertically oriented MXene structures facilitate rapid ion diffusion yet often exhibit reduced mechanical integrity and electrical conductivity. Future research should focus on developing Janus MXene orientation architectures, in which horizontally and vertically oriented MXene domains are spatially integrated within a single structure. By precisely controlling the spatial distribution of these two orientations to achieve a Janus‐oriented structure, the advantages of vertical and horizontal orientations can be synergistically combined while mitigating their respective drawbacks. Such hybrid orientation engineering could enable the MXene device to simultaneously exhibit efficient charge transport, ion accessibility, and mechanical robustness, opening new possibilities for integrated sensing, energy, and electronic applications. In addition, the intrinsic difference in deformation behavior between horizontally and vertically oriented MXene domains provides an opportunity to construct Janus‐oriented architectures capable of producing large, programmable macroscopic deformations, which offers a promising pathway for developing advanced soft MXene‐based actuators for soft robotics and intelligent information systems.

On‐chip compatible, low‐thickness, and high‐density vertical orientation of MXene flakes (Figure 18b). Currently, several techniques, including the directional freezing method, electrical/magnetic‐field‐assisted orientation, and microfluidic spinning, have been developed to achieve vertically oriented MXene architectures. Despite these advances, the vertical orientation engineered from these methods still suffers from some inherent limitations, such as sparse flake stacking, unshrinkable structural dimensions, and restricted architectural design freedom. As a result, most vertically oriented MXene assemblies reported are confined to large‐size bulk and porous forms (e.g., aerogels and scaffolds), which can be beneficial for applications such as energy storage and water treatment, but their large feature dimensions and low packing density make them difficult to employ in on‐chip electronic systems, which demand compact, low‐roughness, and precisely defined structures. Future studies should focus on achieving wafer‐scale, low‐thickness, and high‐density vertical orientation of MXene flakes with precise control over interflake spacing and orientation uniformity. Realizing such dense and nanoscale vertical ordering would mark a critical step toward integrating vertically oriented MXene flakes into thin‐film device architectures compatible with transistors, memristors, and sensors.

Orientation engineering of MXene flakes in MXene/polymer composites (Figure 18c). Although MXene itself exhibits outstanding electrical conductivity, mechanical strength, and intrinsic responsiveness, incorporating them into polymer composites can offer an even more intriguing combination of properties. Within polymer matrices, the rheological behavior and interfacial interactions can be tailored to guide the orientation of MXene flakes during various fabrication processes, including solution casting, stretching, printing, or electrospinning. Moreover, incorporating polymers can diversify the mechanical characteristics of MXene‐based structures, enabling tailored combinations of strength, flexibility, and adaptability. Rigid polymers such as Kevlar can significantly enhance the tensile strength and toughness of MXene architectures through strong interfacial interactions and efficient stress transfer, resulting in mechanically robust composites suitable for structural or load‐bearing applications. In contrast, flexible or stimuli‐responsive polymers, such as PVA and liquid crystal elastomers, can impart high deformability and reversible actuation capability, allowing MXene composites to undergo large, recoverable shape transformations under external stimuli, including heat, humidity, or electric fields. Additionally, polymer composites can serve as an encapsulation barrier, effectively protecting MXene flakes from environmental degradation and mitigating oxidation, hydrolysis, and structural delamination during storage or operation. Therefore, orientation engineering in MXene/polymer composites will stand as a crucial pathway for advancing next‐generation MXene‐based materials and devices with enhanced stability, flexibility, and functionality.

Advanced characterization techniques for visualizing the evolution of MXene orientation (Figure 18d). Currently, a range of techniques, including XRD, WAXS, GIWAXS, polarized Raman spectroscopy, and electron microscopy, are employed to characterize MXene orientation. However, the majority of current studies rely on ex situ measurements. Although these techniques are capable of revealing orientation information at specific temporal or spatial states, they provide only discrete snapshots of the system rather than a continuous picture of how MXene flakes evolve and reorganize during structure assembly or device operation. To obtain a more accurate and comprehensive understanding, it is crucial to develop in situ characterization techniques capable of tracking the reorientation behaviors of MXene flakes during the orientation engineering process. In parallel, the preparation of intermediate states during the MXene orientation process is equally critical. In many cases, the intermediate assemblies exist in a wet or semisolid state, where solvent interactions and interflake spacing play decisive roles in determining the final orientation. However, conventional sample preparation procedures, such as drying in ambient air or an oven, can significantly alter or even disrupt the original orientation of MXene flakes, leading to misleading orientation information. Therefore, careful attention must be paid to preserving the native MXene orientation during characterization. For example, employing rapid quenching techniques, cryogenic preservation, or direct observation under cryo‐SEM may be a suitable choice for accurate and intuitive analysis of MXene orientation.

Computational modeling and theoretical insights into MXene orientation engineering (Figure 18e). At present, computational investigations in MXene orientation research remain relatively limited, despite the rapid progress achieved experimentally. Most existing studies rely primarily on empirical observations or phenomenological models, while systematic simulations that capture the dynamics of flake orientation and interfacial interactions are still scarce. Future work should aim to develop multiscale computational frameworks that bridge atomistic, mesoscale, and continuum levels to describe orientation evolution across different time and length scales. For instance, combining coarse‐grained molecular dynamics or dissipative particle dynamics with phase‐field or computational fluid dynamics can realistically simulate the flow‐induced orientation process in solution or polymer matrices. In parallel, machine‐learning‐assisted modeling can accelerate the mapping between theoretical structural parameters and experimentally observed orientation factors. Integrating these computational insights with in situ characterization data will enable a predictive, quantitative understanding of MXene orientation mechanisms, guiding the rational design of orientation‐engineered MXene architectures in practical device scenarios.

Toward engineering the in‐plane orientation of MXene flakes (Figure 18f). While tremendous progress has been made in the orientation engineering of MXene flakes, most current strategies have focused on regulating their out‐of‐plane orientation, such as controlling the stacking or tilting of flakes. Controlling their in‐plane rotational orientation, where all basal planes are oriented along a common azimuthal direction, has thus far proven nearly unattainable, possibly owing to the weak in‐plane anisotropic interactions and the lack of a strong directional driving force during solution‐based assembly. Nevertheless, the prospect of achieving in‐plane orientation is appealing. If such in‐plane orientation coherence could be achieved, MXene assemblies would evolve beyond conventional out‐of‐plane oriented structures to behave as quasi‐single‐crystalline materials, exhibiting greatly reduced interflake resistance and scattering. Such quasi‐single‐crystalline MXene structures could revolutionize a broad range of disciplines, including microelectronics, photonic and plasmonic engineering, and quantum information technologies. Therefore, although achieving in‐plane orientation of MXene flakes remains highly challenging, continued exploration in this direction is strongly encouraged.

Beyond the emerging research directions discussed above, looking forward, translating MXene orientation engineering from laboratory‐scale demonstrations to large‐scale manufacturing will be equally important. Still, several practical challenges remain for the large‐scale fabrication of orientation‐engineered MXene architectures. First, scalable fabrication of oriented MXene architectures mostly relies on MXene dispersions or inks. While dispersion preparation and handling are easily controllable at the laboratory scale, maintaining its stability during storage and processing becomes difficult upon scale‐up. These stability challenges typically span three dimensions: chemical stability (against oxidation), colloidal stability (against aggregation, restacking, and sedimentation), and rheological stability (against viscosity drift). In this regard, optimizing dispersion chemistry (e.g., tuning solvent composition and employing trace stabilizers) can be useful for improving the multidimensional stability of the dispersion, thereby enhancing process controllability in the large‐scale fabrication of oriented MXene architectures. Second, except for MXene hydrogel, most oriented MXene architectures require a postfabrication drying step. Upon scale‐up, shrinkage stresses and edge effects become more pronounced, making it difficult to preserve orientation uniformity during drying for large‐area fabrication. Possible mitigation routes include designing dedicated manufacturing setups, such as controlled pressing modules and edge‐management fixtures, to ensure spatially uniform drying at large scale, thereby improving orientation uniformity over large areas. Third, for large‐scale fabrication, it is equally important to preserve the oriented architecture after production, during handling, storage, and subsequent device integration. Oriented MXene assemblies, especially freestanding films and fibers, may undergo relaxation, interlayer slippage, or microcrack formation under bending, stacking, or humidity exposure, leading to partial loss of the designed orientation and possible performance degradation. To address this issue, practical directions include developing advanced storage templates with mechanical supports for large‐area oriented MXene architectures, as well as adopting appropriate encapsulation after device integration to minimize mechanical disturbance or rehydration. Overcoming these barriers is critical for moving oriented MXene architectures from proof‐of‐concept demonstrations toward practical application in the real world.

Overall, orientation engineering of MXene flakes is an emerging yet rapidly expanding discipline, already drawing intense research interest and expected to shape the next stage of MXene science. This review presents a comprehensive and systematic summary of the fundamental principles, current advances, and future outlook in orientation engineering of MXene flakes. We believe it can serve as both a guide for newcomers entering the field and a reference for experienced researchers, while further inspiring continued progress and innovation in this rapidly evolving area of MXene research.

## Funding

This work was supported by King Abdullah University of Science and Technology (KAUST).

## Conflicts of Interest

The authors declare no conflicts of interest.

## Data Availability

The data that support the findings of this study are available from the corresponding author upon reasonable request.
